# Exploring the Supramolecular Interactions and Thermal Stability of Dapsone:Bipyridine Cocrystals by Combining Computational Chemistry with Experimentation

**DOI:** 10.1021/acs.cgd.3c00387

**Published:** 2023-05-03

**Authors:** Florian Racher, Tom L. Petrick, Doris E. Braun

**Affiliations:** Institute of Pharmacy, University of Innsbruck, Innrain 52c, Innsbruck 6020, Austria

## Abstract

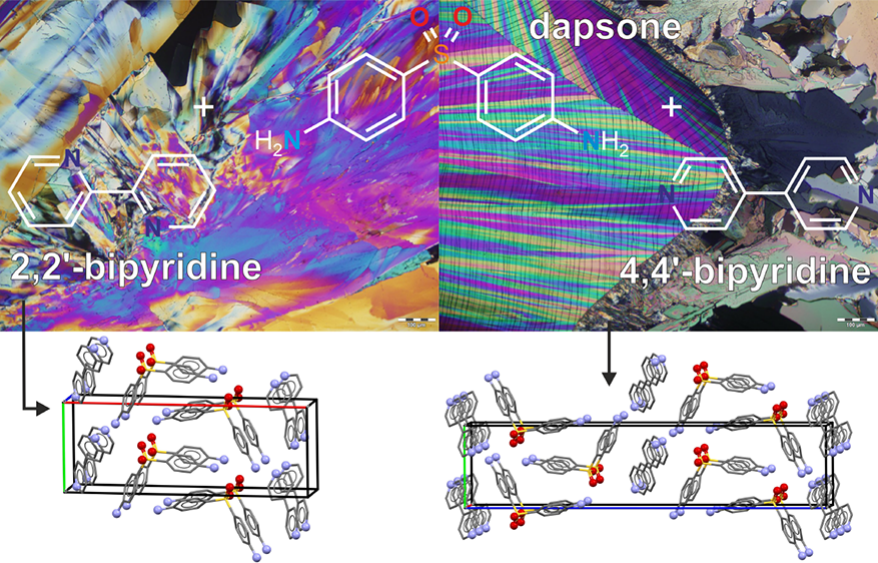

The application of computational screening methodologies based on H-bond propensity scores, molecular complementarity, molecular electrostatic potentials, and crystal structure prediction has guided the discovery of novel cocrystals of dapsone and bipyridine (DDS:BIPY). The experimental screen, which included mechanochemical and slurry experiments as well as the contact preparation, resulted in four cocrystals, including the previously known DDS:4,4′-BIPY (2:1, CC_44_-B) cocrystal. To understand the factors governing the formation of the DDS:2,2′-BIPY polymorphs (1:1, CC_22_-A and CC_22_-B) and the two DDS:4,4′-BIPY cocrystal stoichiometries (1:1 and 2:1), different experimental conditions (such as the influence of solvent, grinding/stirring time, etc.) were tested and compared with the virtual screening results. The computationally generated (1:1) crystal energy landscapes had the experimental cocrystals as the lowest energy structures, although distinct cocrystal packings were observed for the similar coformers. H-bonding scores and molecular electrostatic potential maps correctly indicated cocrystallization of DDS and the BIPY isomers, with a higher likelihood for 4,4′-BIPY. The molecular conformation influenced the molecular complementarity results, predicting no cocrystallization for 2,2′-BIPY with DDS. The crystal structures of CC_22_-A and CC_44_-A were solved from powder X-ray diffraction data. All four cocrystals were fully characterized by a range of analytical techniques, including powder X-ray diffraction, infrared spectroscopy, hot-stage microscopy, thermogravimetric analysis, and differential scanning calorimetry. The two DDS:2,2′-BIPY polymorphs are enantiotropically related, with form B being the stable polymorph at room temperature (RT) and form A being the higher temperature form. Form B is metastable but kinetically stable at RT. The two DDS:4,4′-BIPY cocrystals are stable at room conditions; however, at higher temperatures, CC_44_-A transforms to CC_44_-B. The cocrystal formation enthalpy order, derived from the lattice energies, was calculated as follows: CC_44_-B > CC_44_-A > CC_22_-A.

## Abstract 2

Virtual coformer screening tools guided the discovery of novel dapsone cocrystals with 2,2′- and 4,4′-bipyridine. Despite being related compounds, different cocrystallization preferences were seen and unraveled using crystal structure prediction combined with X-ray diffraction, IR spectroscopy, and thermal analysis.

## 1 Introduction

The use of multicomponent phase systems, such as cocrystals, allows to modify and improve critical physicochemical properties of active pharmaceutical ingredients (APIs), i.e., chemical/physical stability and solubility,^1−3^ without compromising their pharmacological effects. However, such efforts require a sound understanding of the chemical and structural features of a molecule (API), the selection of suitable molecular partners (coformer), and a versatile methodology.^4^ In 2018, the US Food and Drug Administration (FDA) guideline “Regulatory Classification of Pharmaceutical Cocrystals” categorized cocrystals as alternative crystal forms of an API, analogous to polymorphs.^5^ This assessment has elicited considerable commercial interest in cocrystals, as it enables drug manufacturers to use existing safety and efficacy data in Abbreviated New Drug Applications or the registration of medicines with new indications. For generic manufacturers, novel solid forms of APIs provide a means of overcoming some of the patent protection afforded to commercialized medicines.^6^ As a result, screening for cocrystals is increasingly becoming an integral step in the development of modern drug products.

The utilization of coformers as functional parts of new solid forms adds complexity to the screening process, as these coformer molecules may have significantly different physicochemical properties compared to an API. Solvent evaporation, solution cooling crystallization, thermal methods,^7,8^ mechanochemical grinding^9,10^ (neat, liquid assisted,^11^ polymer additive grindning^12^), spray and freeze drying,^13^ and hot-melt extrusion are methods that have been successfully applied in cocrystal screens. A range of virtual screening methods for cocrystallization have evolved. Frequently applied methods are based on predicting the possible interactions between the molecules and coformers,^14^ i.e., specific criteria based on electrostatic potentials between the molecules involved,^15−18^ models trained from large crystallographic databases for predicting the propensity of competing H-bond donor and acceptor interactions,^19−22^ and approaches involving molecular complementarity.^23^ Furthermore, liquid-phase models have been adapted to estimate the mixing enthalpies between coformers^24^ and approaches that are based on the Hansen solubility approach.^25^ Several models based on molecular descriptors combined with machine learning have also been proposed.^26−29^ However, none of these methods considers the crystal environment explicitly and its effects on the stability of a proposed cocrystal. Crystal structure prediction (CSP) methods, whose applicability and reliability have increased significantly over the last decades, address exactly the latter. CSP on cocrystals is challenging, because increasing the number of molecules in the crystal’s asymmetric unit comes along with an increase in the number of degrees of freedom that need to be explored in identifying all low-energy crystal structures. Nevertheless, CSP has been shown to be invaluable in predicting cocrystal formation and accessing cocrystal structures.^30−36^

**Figure 1:**
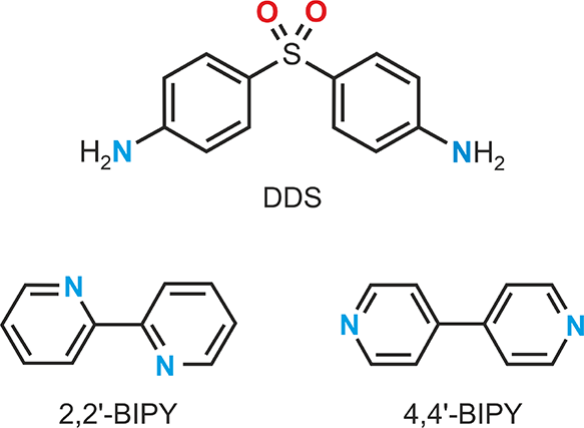
Molecular diagrams of dapsone (DDS), 2,2′-bipyridine, and 4,4′-bipyridine (BIPY).

In this work, we started our cocrystal screening process by using the hydrogen-bond propensity tool to investigate cocrystal formation of the anti-infective agent dapsone (DDS, Figure 1). Selected coformers were then subjected to molecular complementarity and molecular energy potential map calculations. Surprisingly, some of the virtual screening tools yielded different outcomes for the two bipyridine isomers (2,2′-BIPY and 4,4′-BIPY). This sparked our interest in investigating cocrystallization of DDS with both 2,2′-BIPY and 4,4′-BIPY.

DDS has been identified by the World Health Organization as an essential medicine in the treatment of leprosy in combination with rifampicin and clofazimine.^37^ Recently, we have investigated the polymorphism^38,39^ and solvate^40,41^ (hydrate^42^) formation of DDS. Cocrystallization of DDS may offer benefits, especially increasing its low water solubility. Several cocrystals have already been reported for DDS, including those with 5-nitroisophthalic acid,^43^ 3,5-dinitrobenzoic acid,^44^ 1,3,5-trinitrobenzene,^45^ 1-(pyridin-4-yl)piperazine,^46^ 4,4′-bipyridine,^47^ ε-caprolactame,^47^ flavone,^48,49^ 3-benzothiazol-2(3*H*)-one,^49^ caffeine,^49^ 1,3,7-trimethyl-3,7-dihydro-1*H*-purine-2,6-dione,^49^ 1,3-benzothiazol-2(3*H*)-one,^49^ 1,3,5,7-tetra-azatricyclo[3.3.1.1^3,7^]decane,^50^ 2-(3,4-dihydroxyphenyl)-5,7-dihydroxy-4*H*-chromen-4-one^49^ (cocrystal ethanol solvate), and a drug–drug cocrystal with sulfanilamide.^49^

Bipyridines are heavily used as ligands in coordination chemistry, as metal chelating ligands and connectors between transition metal atoms for the propagation of coordination networks.^51−54^ Furthermore, the amino groups make bipyridines an interesting class of molecules for cocrystal formation.^55−61^ In our recent work, we focused on solvate formation of the two BIPYs.^62^ Interestingly, different tendencies toward the formation of multicomponent solid-state forms with carboxylic acids were observed. The more exposed location of the N atoms in the 4,4′-BIPY seems to favor the formation of carboxylic acid solvates. Hence, we tested different experimental conditions for the formation of DDS:BIPY cocrystals, focusing on mechanochemical reactions, slurry experiments in water and organic solvents, and the contact preparation method. The influence of the solvent, time (grinding, slurry experiments), and molar ratios of the starting materials (DDS:BIPY) on the cocrystallization tendency was investigated and contrasted to the virtual screening results. Thus, a more complete picture of DDS:BIPY cocrystals is provided and benefits and limitations of experimental and virtual screening approaches are discussed.

## 2 Materials and Methods

### 2.1 Virtual Cocrystal Screening

#### 2.1.1 Multicomponent Hydrogen-Bond (MCHB) Propensity Screen

A set of 116 potential coformers (listed in the Supporting Information, Table S1) was selected and ranked based on their MCHB scores, which were calculated using mercury.^63^ The propensity of the highest heteromeric interaction between DDS and a coformer (*P*_D–C_) was compared with the highest homomeric interaction, either DDS–DDS (*P*_D–D_) or coformer–coformer (*P*_C–C_). The difference, Δ_HBP_ = *P*_D–C_ – [max(*P*_D–D_, *P*_C–C_)], was used to estimate the likelihood of cocrystallization, with higher values indicating greater likelihood of cocrystallization.

#### 2.1.2 Molecular Complementarity (MC) Screen

The 20 coformers with the highest MCHB propensity scores were selected for further analysis using the MC search method (Supporting Information, Table S2).^23,63^

#### 2.1.3 Molecular Electrostatic Potential (MEP) Maps

The MEPs of DDS, 2,2′-BIPY, and 4.4′-BIPY were mapped onto their 0.002e^–^ Å^–3^ electron density isosurface, which was computed by Gaussian 16^64^ using the B3LYP DFT method and 6-311++G(d,p) basis set. The local maxima (MEP_max_) and minima (MEP_min_) were identified and used to calculate the H-bond donor parameter (α) and H-bond acceptor parameter (β), using eqs 1 and 2, respectively, as described in previous studies.^14^
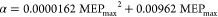
1
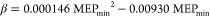
2

The total pairing energy in the solid state was estimated as the sum over a hierarchical listing of a specific number of complementary H bond donor–acceptor sites (3):
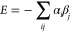
3

The possible energy gain (Δ*E*, kJ mol^–1^) upon cocrystal formation was calculated according to eq. 4:
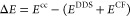
4

CC, DDS, and CF represent the cocrystal, dapsone, and coformer, respectively, and the energies correspond to the interaction energies calculated from the α and β values.

### 2.2 Computational Generation of the Single- and Multicomponent Crystal Energy Landscapes

The crystal energy landscapes of DDS:2,2′-BIPY (1:1) and DDS:4,4′-BIPY (1:1) were calculated using the same procedure applied to generate the single-component crystal energy landscpaes.^39,62^ Rigid-body CrystalPredictor v2^65−67^ searches were performed in the 59 most common space groups (*Z*′ = 1), and an additional *Z*′ = 2 search was performed for DDS and 2,2′-BIPY in *P*2_1_ only. The global minimum conformations were chosen for DDS^39^ and 2,2′-BIPY, while for 4,4′-BIPY, the minimum conformation and a maximum were chosen. The local maximum corresponds to the most frequent conformation seen among all CSD structures and is only 6 kJ mol^–1^ less stable than the global minimum.^62^ The low-energy structures (30 kJ mol^–1^ range with respect to the global minimum structure) were reoptimized with DMACRYS^68^ and then with CrystalOptimizer v2.4.8^69^ using a distributed multipole representation of the charge density^70^ (DDS:2,2′-BIPY: 40 kJ mol^–1^ range; DDS:4,4′-BIPY: 30 kJ mol^–1^ range). The conformational energies and distributed multipoles used were calculated at the PBE0/6-31G(d,p) and PBE0/aug-cc-pVTz levels using Gaussian09,^71^ respectively, and all other intermolecular forces were modeled in an atom–atom *exp-6* form using the FIT potential.^68,72^

The most stable cocrystal structures (DDS:2,2′-BIPY: 25 kJ mol^–1^ range; DDS:4,4′-BIPY: 20 kJ mol^–1^ range) were then optimized with CASTEP v20.11^73^ using the PBE generalized gradient approximation (GGA) exchange-correlation density functional^74^ and ultrasoft pseudopotentials,^75^ with the addition of the Tkatchenko and Scheffler (TS)^76^ semiempirical dispersion. The number of *k*-points was chosen to provide a maximum spacing of 2π · 0.07 Å^–1^, and a basis set cut-off of 780 eV was used. Convergence criteria were as follows: <2 × 10^–5^ eV per atom, atomic displacements <1 × 10^–3^ Å, maximum forces <5 × 10^–2^ eV Å^–1^, and maximum stresses <0.1 GPa.

The energies of the final crystal energy landscapes were then recalculated using CASTEP and the MBD* dispersion correction,^77^ all other settings were the same as described for the PBE-TS optimizations. The set of structures included all computationally generated DDS,^39^ 2,2′-BIPY, 4,4′-BIPY,^62^ and cocrystal low-energy structures (25 kJ mol^–1^ range).

The cocrystal formation enthalpies (ΔΔ*E*_*F*_^CC^) were calculated according to eq 5,
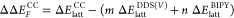
5where Δ*E*_latt_ corresponds to the lattice energy and *m* and *n* to the number of DDS and coformer molecules present in the cocrystal.

Finally, COMPACK^78^ and the CCDC API packing similarity dendrogram script were used for clustering the structures.

### 2.3 Pairwise Intermolecular Energy Calculations

To calculate the pairwise intermolecular energy,^79−81^ we used CrystalExplorer V17,^82^ along with the PBE-MBD* optimized structures and a 3.8 Å radius. Gaussian16^64^ was used to calculate B3LYP/6-31G(d,p) molecular wave functions, from which the densities of unperturbed monomers were derived to obtain the four distinct energy components: electrostatic (*E*_E_), polarization (*E*_P_), dispersion (*E*_D_), and exchange repulsion (*E*_R_).

### 2.4 Experimental Cocrystal Screen

#### 2.4.1 Materials

Dapsone (form III, purity 97%) and 4,4′-BIPY (hydrate, ≥98%) were purchased from Aldrich; 2,2′-BIPY (≥99%) from Sigma-Aldrich. Dapsone was recrystallized from a hot-saturated methanol solution (form III), and the 4,4′-BIPY hydrate was dehydrated at 0% RH (over P_2_O_5_). 2,2′-BIPY was used as obtained.

#### 2.4.2 Dry and Liquid-Assisted Grinding Experiments

Mixtures of 1:1, 1:2, and 2:1 (DDS:BIPY) molar ratios were prepared. To 150 mg of the mixture, 5 (water) or 10 drops of solvent [*t*-butanol, *i*-butyl acetate, diispropyl ether (DIPE), or *n*-heptane] were added, and the resulting paste was milled in stainless steel vessels with three balls of the same material and 0.5 cm in diameter using a Retsch ball mill MM 2000 (Haan, Germany) at 15 Hz for 60 min. For dry grinding experiments, 200 mg of the mixture was used. Samples were periodically withdrawn (after 5, 10, 15, 30, 45, and 60 min) and analyzed using IR spectroscopy and PXRD.

#### 2.4.3 Slurry Experiments in Organic Solvents

Mixtures of 1:1, 1:2, and 2:1 molar ratios were prepared and transferred to small vials. To 150 mg of the mixture, 200–500 μL of solvent was added, and the slurry was stirred in a cycling temperature range from 10 to 30 °C. Samples were periodically withdrawn and analyzed using PXRD and IR spectroscopy.

#### 2.4.4 Contact Preparation

The contact preparation method^83,84^ was used to quickly discriminate between eutectic and cocrystal formation, provided the components are meltable and sufficiently thermally stable.^85^ First, the higher-melting compound is melted on a microscopic slide covered by a glass slide. After cooling, the lower-melting compound is then melted on the same microscopic slide. The liquid is drawn below the cover slide until it reaches the higher-melting coformer and then cooled. An Olympus BH2 polarization microscope (Olympus Optical GmbH, Vienna, Austria) equipped with a Kofler hot stage (Reichert Thermovar, Vienna, Austria) and an Olympus DP71 digital camera was used.

### 2.5 Synthesis of Cocrystals

#### 2.5.1 DDS:2,2′-BIPY A (1:1)—CC_22_-A

A saturated solution of DDS and 2,2′-BIPY was prepared in water at RT. Then, DDS (614.14 mg) and 2,2′-BIPY (386.39 mg) were added to the solution. The suspension was stirred for five days in the temperature range of 10–30 °C, resulting in CC_22_-A. The product was filtered and dried at RT.

#### 2.5.2 DDS:2,2′-BIPY B (1:1)—CC_22_-B

A saturated solution of DDS and 2,2′-BIPY was prepared in *n*-heptane at RT. DDS (613.44 mg) and 2,2′-BIPY (386.15 mg) were then added to this solution. The suspension was stirred for one month in the temperature range of 10–30 °C. Samples were withdrawn periodically and analyzed using PXRD. The product consisted of CC_22_-B and 2,2′-BIPY impurities. The mixture (approx. 150 mg) was stored at 75 °C for 1 h to remove 2,2′-BIPY, resulting in phase pure CC_22_-B.

#### 2.5.3 DDS:4,4′-BIPY A (1:1)—CC_44_-A

DDS (613.13 mg) and 386.13 mg of 4,4′-BIPY were added to a saturated solution of DDS and 4,4′-BIPY in DIPE (25 °C). The suspension was then stirred between 10 and 30 °C. Cocrystal formation (CC_44_-A) occurred after one day. The product was filtered and dried at RT.

#### 2.5.4 DDS:44′-BIPY B (2:1)—CC_44_-B

Similarly, for the CC_44_-B preparation, a saturated solution of DDS and 4,4′-BIPY was prepared in water at RT. DDS (760.86 mg) and 239.45 mg of 4,4′-BIPY were added to the solution and stirred for four days in between 10 and 30 °C, leading to the formation of CC_44_-B. The product was filtered and dried at RT.

### 2.6 Powder X-Ray Diffraction (PXRD) and Structure Solution from PXRD

PXRD patterns were recorded using an X’Pert PRO diffractometer (PANalytical, Almelo, NL) in transmission geometry, with a Cu-K_α1,2_ radiation source, a PIXcel1D detector, 40 kV/40 mA, and a step size of 2θ = 0.013° with 40 s in the 2θ range from 2° to 40° or a step size of 2θ = 0.007° with 1600 s in the 2θ range from 2° to 70° (structure solution only).

The diffraction patterns of CC_22_-A and CC_44_-A were indexed to a monoclinic unit cell using the first 20 peaks with DICVOL, and the space group was determined to be *P*2_1_ based on a statistical assessment of systematic absences,^86^ as implemented in the DASH structure solution package.^87^ From the cell volume, it was determined that there are two DDS and two 2,2′-BIPY (CC_22_-A), and one DDS and one 4,4′-BIPY (CC_44_-A) in the asymmetric unit. The data were background subtracted, and Pawley refinement^88^ was used to extract the intensities and their correlations. Simulated annealing was used to optimize the models against the diffraction data set in direct space. The internal coordinate (Ζ-matrix) descriptions were derived from the PBE0/6-31G(d,p) gas-phase global conformational minima, with O–H distances normalized to 0.9 Å and C–H distances to 0.95 Å. Each of the structures was solved using 100 simulated annealing runs of 1 × 10^8^ moves per run in DASH. Each DDS molecule was allowed 6 external and 2 internal degrees of freedom, and each BIPY was allowed 6 external and 1 internal degree of freedom. The best solutions (three for CC_22_-A and one for CC_44_-A) were then subjected to PBE-MBD* optimizations (CASTEP). All chosen structure solutions had refined to a χ^2^ ratio of <3.8 (profile χ^2^/pawley χ^2^). The optimized structures with O–H distances normalized to 0.9 Å and C–H distances to 0.95 Å were then used as the starting point for rigid-body Rietveld refinements^89^ using TOPAS V7.12.^90^ The background was modeled with Chebyshev polynomials. The final refinement of CC_22_-A included 67 parameters (22 profile, 4 cell, 1 scale, 1 *U*_iso_, 15 preferred orientation, 12 position, and 12 rotation), resulting in a final *R*_wp_ of 5.98%. For CC_44_-A, the final refinement included 55 parameters (22 profile, 4 cell, 1 scale, 1 *U*_iso_, 15 preferred orientation, 6 position, and 6 rotation) and resulted in a final *R*_wp_ of 3.92%.

### 2.7 Infrared Spectroscopy

Infrared spectra were recorded with a diamond ATR (PIKE GaldiATR, Madison, US) crystal on a Bruker Vertex 70 FTIR spectrometer (Bruker Analytische Messtechnik GmbH, Germany). The spectra were recorded between 4000 and 400 cm^–1^ at an instrument resolution of 2 cm^–1^, with 32 scans per spectrum.

### 2.8 Thermal Analysis and Isothermal Calorimetry

#### 2.8.1 Differential Scanning Calorimetry (DSC)

DSC measurements were performed using a DSC7 (Perkin-Elmer, Norwalk, Connecticut, USA), controlled by the Pyris 8.0 software. Approx. 2–3 mg of sample was weighed into sealed aluminum pans using a UM3 ultramicrobalance (Mettler, Greifensee, Switzerland). Heating rates of 5 and 10 °C min^–1^ were applied, and dry nitrogen was used as a purge gas (20 mL min^–1^). The instrument was calibrated for temperature with pure benzophenone (mp 48.0 °C) and caffeine (236.2 °C), and the energy calibration was performed with indium (mp 156.6 °C, heat of fusion 28.45 J g^–1^). The stated (extrapolated onset) temperatures and enthalpy values have an error calculated at 95% CI and are based on at least three measurements.

#### 2.8.2 Thermogravimetric Analysis (TGA)

TGA was performed using a TGA7 system (Perkin-Elmer, Norwalk, CT, USA) and the Pyris 8.0 Software. Approximately 3 mg of sample was weighed into a platinum pan. Two-point calibration of the temperature was performed with ferromagnetic materials (Alumel and Ni, Curie-point standards, Perkin-Elmer). Heating rates of 1, 2, 5, and 10 °C min^–1^ were applied, and dry nitrogen was used as a purge gas (sample purge: 20 mL min^–1^, balance purge: 40 mL min^–1^).

## 3 Results and Discussion

### 3.1 Virtual Cocrystal Screen

#### 3.1.1 Multicomponent Hydrogen-Bond Propensity

This tool is a knowledge-based method. It analyzes the occurrence of specific intermolecular interactions of a given functional group in the CSD and assumes that the strongest H-bond among all possible donor-acceptor pairs guides the formation of a crystal structure.^19−22^ The multicomponent hydrogen-bond propensity score was calculated for 116 combinations of DDS and coformers (Table 1 and Table S1, the Supporting Information for the full set).

**Table 1:** Selection of Multicomponent Hydrogen-Bond Propensity Screen Results Expressed as the Highest Propensity toward the Formation of Heterodimeric (D:C or C:D)^a^ or Homodimeric (D:D or C:C)^a^ Hydrogen Bond Interactions

ran*k*	coformer (C)	multicomponent score	max interaction	max D:C or C:D propensity	max D:D propensity	max C:C propensity
1	*DL*-malic acid	0.34	C:D	0.93	0.59	0
2	3,5-dihydroxybenzoic acid	0.25	C:D	0.91	0.66	0.6
3	4,4′-bipyridine	0.23	C:D	0.86	0.64	0
4	catechol	0.23	C:D	0.90	0.67	0
4	succinic acid anhydride	0.23	C:D	0.92	0.69	0
6	pyrazine	0.22	C:D	0.86	0.64	0
7	3-methylpyridine	0.21	C:D	0.85	0.64	0
8	phthalic acid anhydride	0.16	C:D	0.85	0.69	0
9	alitame	0.15	C:D	0.80	0.64	0.66
10	orotic acid	0.15	C:D	0.82	0.67	0.61
11	*t*-butylamine	0.15	C:D	0.79	0.63	0.63
12	2,2′-bipyridine	0.14	C:D	0.78	0.64	0
13	camphor	0.14	C:D	0.79	0.65	0
14	4-acetamidobenzoic acid	0.13	C:D	0.85	0.71	0.72
15	nicotinic acid	0.13	C:D	0.84	0.71	0.64

a D—dapsone, C—coformer.

The estimated multicomponent scores ranged from +0.34 to −0.30. Overall, 61 DDS:coformer combinations resulted in a positive value, 8 were calculated as 0, and 43 combinations resulted in a negative value. Out of the list of 116 coformers, 7 received a multicomponent score with DDS well above 0, i.e., *DL*-malic acid, 3,5-dihydroxybenzoic acid, 4,4′-bipyridine, catechol, succinic acid anhydride, pyrazine, and 3-methylpyridine. Interestingly, four out of the seven coformers only feature H-bonding acceptor groups. This observation agrees with the DDS literature cocrystals. The DDS:4,4′-BIPY combination was among the highest ranked coformers featuring no H-bonding donor groups (a multicomponent score of 0.23). 2,2′-BIPY showed a lower multicomponent score with DDS (0.14), but the analysis still predicted cocrystal formation with both isomers.

#### 3.1.2 Molecular Complementarity Screening

The second tool used for cocrystal prediction relies on the shape and polarity of the molecules.^23^ Geometrical descriptors used are the M/L ratio, S, and S/L ratio, where S, M, and L are the lengths of the shortest, medium, and longest axes of a rectangular box enclosing the van der Waals volume of a molecule. MC also compares the dipole moment magnitude and fraction of N and O atoms of the considered molecules. The results of the MC screening will depend (to some extent) on the conformation of the considered molecules,^16^ but for DDS, changes to its conformation are limited and hardly alter the S, M, L parameters.

Out of the 20 best MCHB coformers, only 8 passed the MC test. None failed the dipole moment magnitude test. However, five failed the fraction of N and O atoms test and nine the geometrical descriptor analyses (Table 2 and Table S2, Supporting Information). The highest ranked coformer passing the MC test was 4,4′-BIPY, which is also known to form cocrystals^47^ with DDS. Surprisingly, 2,2′-BIPY failed due to slightly different geometrical values, i.e., planar (optimized geometry, affecting the S value) compared the predominantly twisted orientation of 4,4′-BIPY (optimized geometry).^62^

**Table 2:** Results of the Molecular Complementarity Search of the Top 20 Ranked Multicomponent Hydrogen-Bond Propensity (MCHB) Coformers

rank (MCHB)	coformer	overall	rank (MCHB)	coformer	overall
1	*DL*-malic acid	FAIL	11	*t*-butylamine	**PASS**
2	3,5-dihydroxybenzoic acid	FAIL	12	2,2′-bipyridine	FAIL
3	4,4′-bipyridine	**PASS**	13	camphor	**PASS**
4	catechol	FAIL	14	4-acetamidobenzoic acid	FAIL
5	succinic acid anhydride	FAIL	15	nicotinic acid	FAIL
6	pyrazine	FAIL	16	ε-caprolactam	**PASS**
7	3-methylpyridine	**PASS**	17	xanthine	FAIL
8	phthalic acid anhydride	FAIL	18	acetic acid	FAIL
9	alitame	**PASS**	19	l-methionine	**PASS**
10	orotic acid	FAIL	20	*N*-ethylacetamide	**PASS**

#### 3.1.3 Molecular Electrostatic Potential Maps

The tool, which is based on electrostatic potential maps, provides an estimate of the energy gain upon possible intermolecular interactions between the two considered molecules.^14,15^ The MEP analysis considers all possible pairs of electrostatic interactions, as for each possible interaction site, the α or β values are calculated (α: H bond-donating sites, β: H bond-accepting). All sites are then paired according to the strength of their interactions, and thus, MEP maps provide an estimate of the energy gain upon cocrystal formation. Similar to the MC analysis, the molecular conformation can influence the obtained results.

**Figure 2:**
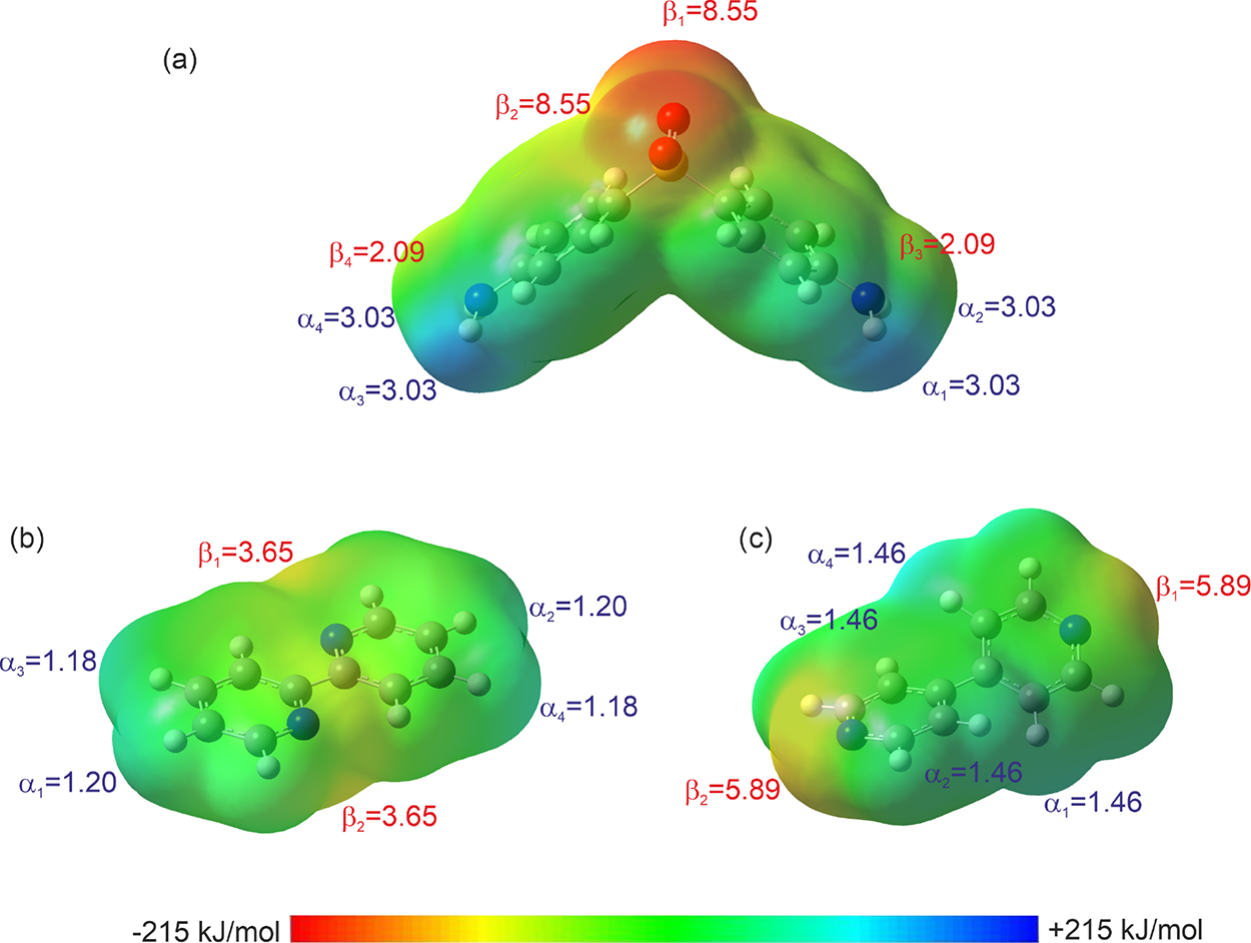
Multicomponent MEP of the gas-phase optimized geometry of (a) DDS, (b) 2,2′-BIPY, and (c) 4,4′-BIPY with the calculated α and β values, representing the strength of the hydrogen bond-donating and hydrogen bond-accepting sites, respectively.

Figure 2 shows the MEP maps together with the respective α and β values obtained for DDS and the two BIPY isomers. As expected, the BIPY maps differ in the location of the strongest β parameter. More importantly (with respect to cocrystallization tendency), the α and especially β values differ for the two coformers. Applying eq 3, energy values of −69.90, −11.51, and −17.73 kJ mol^–1^ were obtained for DDS, 2,2′-BIPY, and 4,4′-BIPY, respectively. This then translated into an energy gain (Δ*E*, eq 4) of −7.83 kJ mol^–1^ for the combination DDS and 2,2′-BIPY and −17.90 kJ mol^–1^ for DDS and 4,4′-BIPY (1:1 ratio). The latter values suggest that both isomers should cocrystallize with DDS. 4,4′-BIPY was estimated to be the better coformer (independent of the input conformation). The latter agrees qualitatively with the MCHB and MC calculations. Modifying the 1:1 ratio of DDS:BIPY to 1:2 or 2:1 barely changes the energy for 2,2′-BIPY (≤0.34 kJ mol^–1^, Table S3, Supporting Information). Thus, no preferred stoichiometric ratio could be derived from the MEP calculations. In case of DDS:4,4′-BIPY, a higher stabilization seemed to be achievable in case of the 1:2 (−20.35 kJ mol^–1^) and 2:1 (−19.18 kJ mol^–1^) molar ratios. Nevertheless, the values are close in energy.

### 3.2 Computational Generation of the Single- and Multicomponent Crystal Energy Landscapes

**Figure 3:**
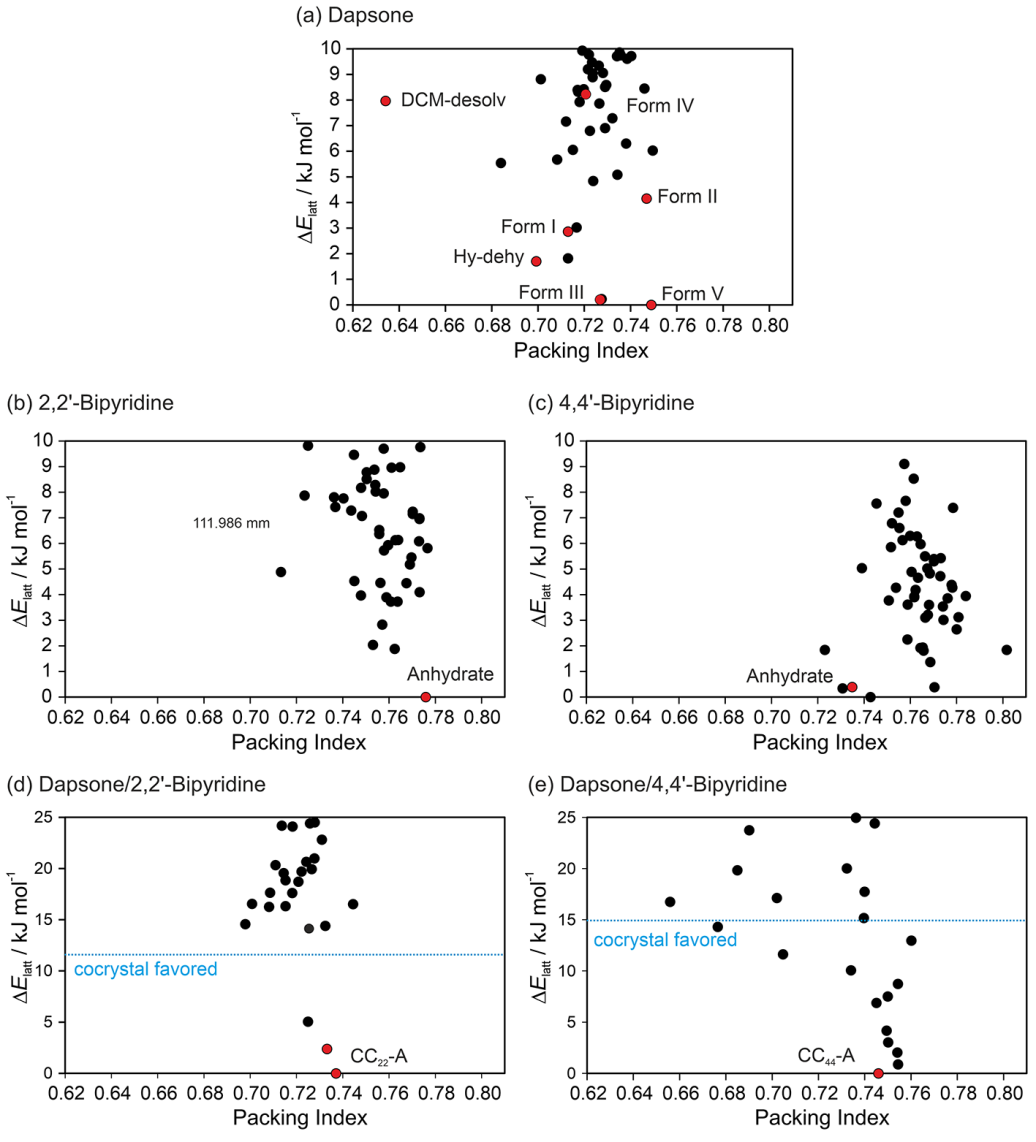
Single- and multicomponent solid-form landscapes of (a) DDS, (b) 2,2′-BIPY, (c) 4,4′-BIPY, (d) DDS:2,2′-BIPY (1:1), and (e) DDS:4,4′-BIPY (1:1). The experimental structures are coded in color and labeled. The blue horizontal line in panels d and e serves as a threshold to distinguish between thermodynamically feasible cocrystal structures and those that are not (based on their calculated lattice energies).

CSP was used to generate the lattice energy landscapes of the single- and multicomponent crystal forms. The previously published lattice energy landscapes for the single-component crystals^39,62^ were reoptimized using the same methodology applied for generating the (1:1) cocrystal landscapes (Figure 3). In the DDS case, the global lattice energy minimum corresponded to the thermodynamic form V (at 0 K and RT). The second most stable structure was form III, the marketed polymorph (metastable form with a very high kinetic stability). All other polymorphs and isostructural dehydrate/desolvate structures were found within 10 kJ mol^–1^ of form V. In case of the coformers, 2,2′-BIPY was found as the global minimum structure and 4,4′-BIPY 0.4 kJ mol^–1^ above the global minimum. Furthermore, the geometries were well reproduced, with RMSD_30_ values below 0.43 Å (DDS and BIPY), suggesting the CSP methodology applied describes the chosen molecules sufficiently.

**Figure 4:**
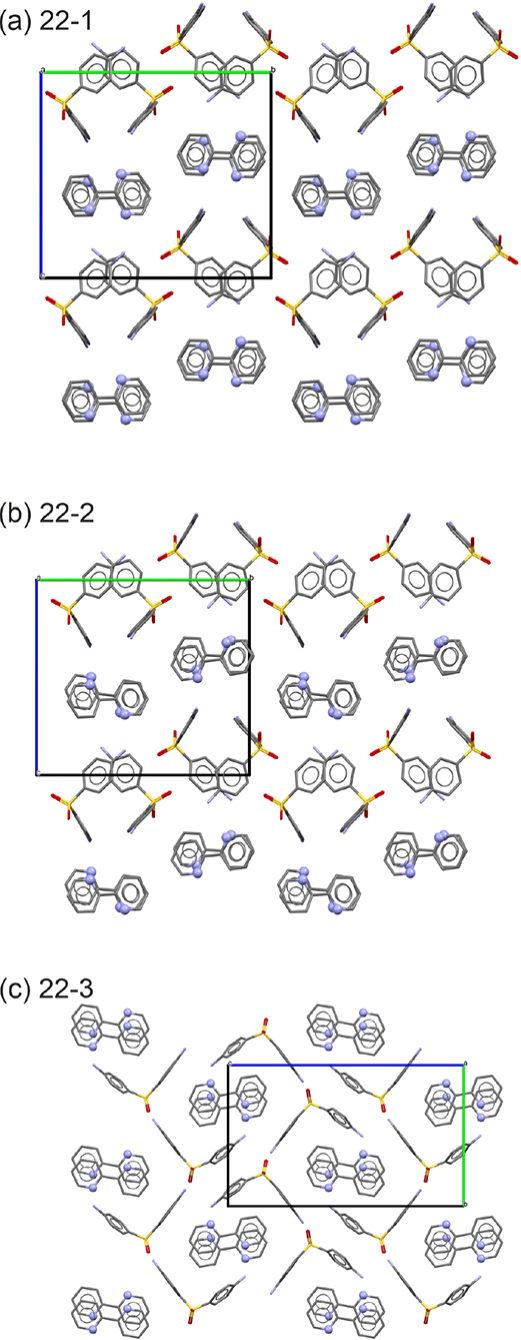
Computationally generated lowest-energy DDS:2,2′-BIPY cocrystal structures. Bipyridine N atoms are denoted as balls, and H atoms are omitted for clarity.

The CSP methodology was used to screen potential BIPY coformers for cocrystallization with DDS in a 1:1 ratio. The cocrystal structures were compared to the lattice energies of the experimental DDS and BIPY structures to identify thermodynamically feasible packings. Initially, only one packing was found for DDS:2,2′-BIPY, which was calculated to be more stable than the sum of the lattice energies of DDS form V and 2,2′-BIPY—structure 22-3 (Table S7, Supporting Information). As suggested by experiments (see the next section), the search was expanded by a monoclinic *P*2_1_*Z*′ = 2 search and three thermodynamically feasible packings were found (Figure 3d). The two lowest energy structures (22-1 and 22-2) are *Z*′ = 2 and differ only in the orientation of one of the two crystallographically independent 2,2′-BIPY molecules (Figure 4a,b) and, therefore, may be two ordered variants of a disordered structure. Interestingly, a third variation of this structure (rank 4) was calculated to be less stable than the sum of DDS and 2,2′-BIPY. The lowest energy structure can be described as a corrugated layer structure, composed of DDS and 2,2′-BIPY layers, with strong inter- and intralayer interactions (H-bonding and aromatic), while the rank 3 structure (Figure 4c) is a 3D network of DDS molecules interlinked through H-bonding and aromatic interactions, accommodating stacks of 2,2′-BIPY molecules that form strong H-bonding interactions to DDS. The *Z*′ = 1 arrangement is approx. 5 kJ mol^–1^ less stable than the global minimum (*Z*′ = 2).

**Figure 5:**
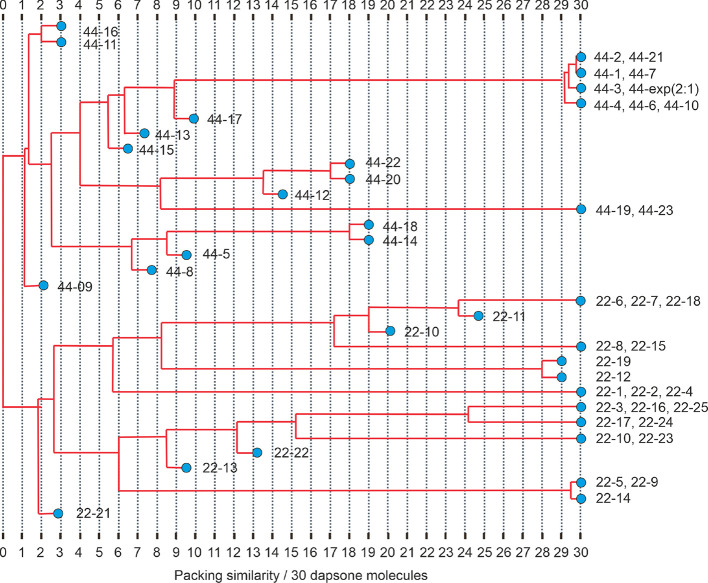
Packing similarity dendrogram based on the computationally generated low-energy cocrystal structures lining clusters based on their average packing similarity. Note that the bipyridine molecules were omitted for the comparisons. 22-*x* and 44-*x* correspond to the computationally generated DDS:2,2′-BIPY and DDS:4,4′-BIPY structures, respectively.

The packing similarity dendrogram in Figure 5 illustrates the structural relationships between the computed DDS:BIPY cocrystal structures. The dendrogram was constructed by comparing clusters of 30 DDS molecules in each packing and omitting the BIPY molecules from the comparison. In case of DDS:2,2′-BIPY, only 5 out of the 25 structures show a unique 3D network of DDS not seen in other packings. This indicates that the 2,2′-BIPY molecules can adopt more than one orientation due to their molecular features (e.g., planarity). The dihedral angle between the two pyridyl rings in the 2,2′-BIPY molecule ranges from 0° to 23°. Hence, BIPY adopts the close to planar orientation in the cocrystal structures that is consistent with the most frequently occurring conformation among all 2,2′-BIPY structures.^62^ A detailed list of the cocrystal structures, including lattice parameters, is provided in Tables S7 and S8, Supporting Information.

**Figure 6:**
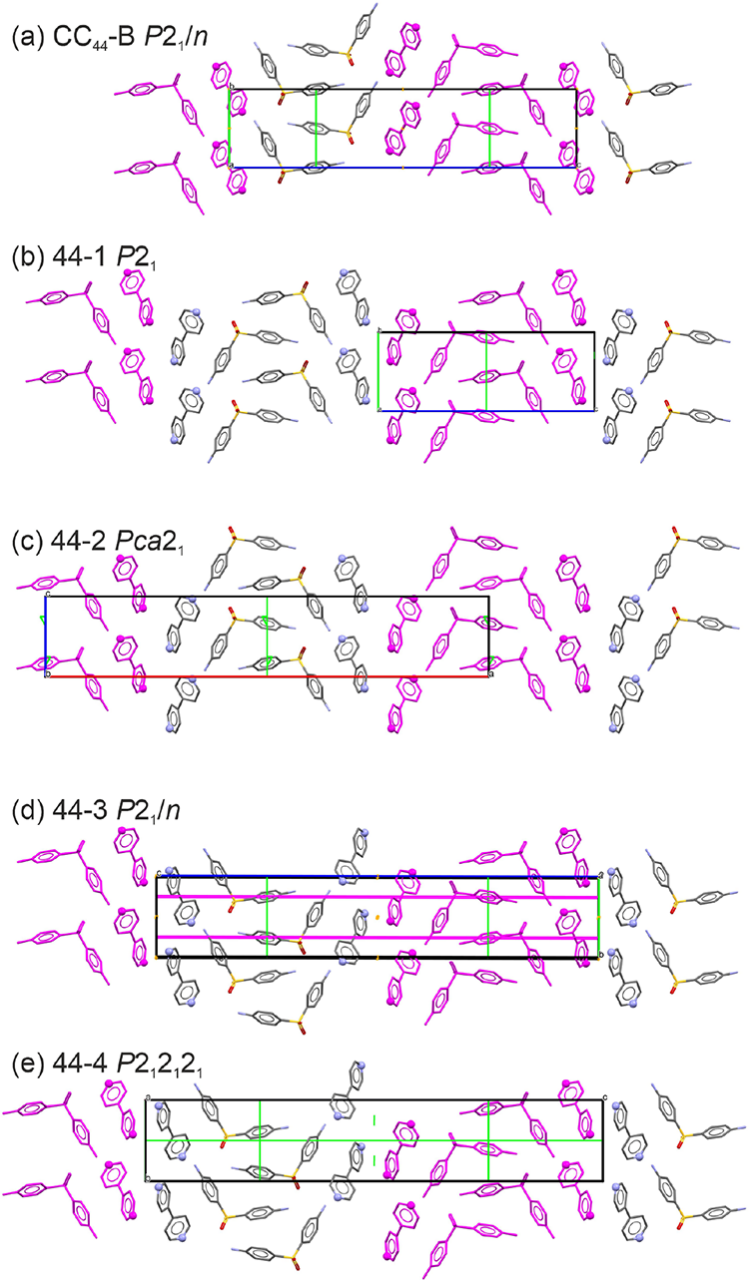
Packing diagrams of (a) the experimental CC_44_-B (2:1), (b) 44-1, (c) 44-2, (d) 44-3, and (e) 44-4 structures. 2D building blocks being present in all structures are color coded, and selected symmetry operations are shown. 44-1 to 44-4 are predicted structures.

The energy landscape of DDS:4,4′-BIPY exhibits several packings that are more stable than the sum of DDS V and 4,4′-BIPY anhydrate lattice energies, which are located below the dashed line on Figure 3e. Since the known cocrystal (CC_44_-B) has a 2:1 stoichiometry, it cannot be found among the 1:1 crystal structures. To explore this further, we compared the packing arrangements of CC_44_-B and the computationally generated low-energy structures (Figure 5). Surprisingly, multiple hypothetical 1:1 structures bear a strong resemblance to CC_44_-B. The cluster shown in Figure 5 in the upper right corner contains the experimental, 7 of the 10 lowest energy structures (incl. the global minimum) and a higher energy structure. A closer inspection of the nine related structures, with a subset shown in Figure 6, revealed that all structures within 8 kJ mol^–1^ of the global minimum exhibit the same 2D building block, which contains 2_1_ and translationally related DDS molecules and 4,4′-BIPY molecules H-bonded to DDS (highlighted in magenta). The difference between the 2:1 and 1:1 structures is that the DDS layers are separated by a single or double layer of BIPY molecules, respectively. Therefore, in the 1:1 structures, only one of the two BIPY N atoms forms a H-bonding interaction, whereas in CC_44_-B, both are involved in strong H-bonding (BIPY lies on an inversion center). The fact that the experimental and low-energy predicted structures are composed of the same 2D building block makes this arrangement highly favorable for DDS:4,4′-BIPY. Adjacent building blocks can be related by translation, glide plane, inversion or 2_1_ screw-axis. Structures 44-1 to 44-4 were calculated to lie within approx. 3 kJ mol^–1^ in lattice energy.

The CSP results suggest that both 2,2′-BIPY and 4,4′-BIPY can cocrystallize with DDS. Based on the calculated cocrystal formation enthalpies (eq 5, Tables S7 and S8), the numbers of potential structures and energy values (ΔΔ*E*_*F*_^CC^) differ for the two isomers. More structures and higher stabilization values were found and calculated for 4,4′-BIPY than 2,2′-BIPY, respectively. Hence, 4,4′-BIPY may be seen as the better DDS cocrystallizing molecule of the two isomers.

### 3.3 Experimental Cocrystal Screen

Mechanochemical formation of cocrystals, slurry experiments in selected solvents, and the contact preparation method were chosen for the experimental cocrystal screen. Classic solution crystallization attempts failed due to solubility differences between DDS and the coformers. The outcome of the screening experiments was evaluated by PXRD, and any potential cocrystal formations were further examined using IR, HSM, DSC, and TGA experiments.

**Figure 7:**
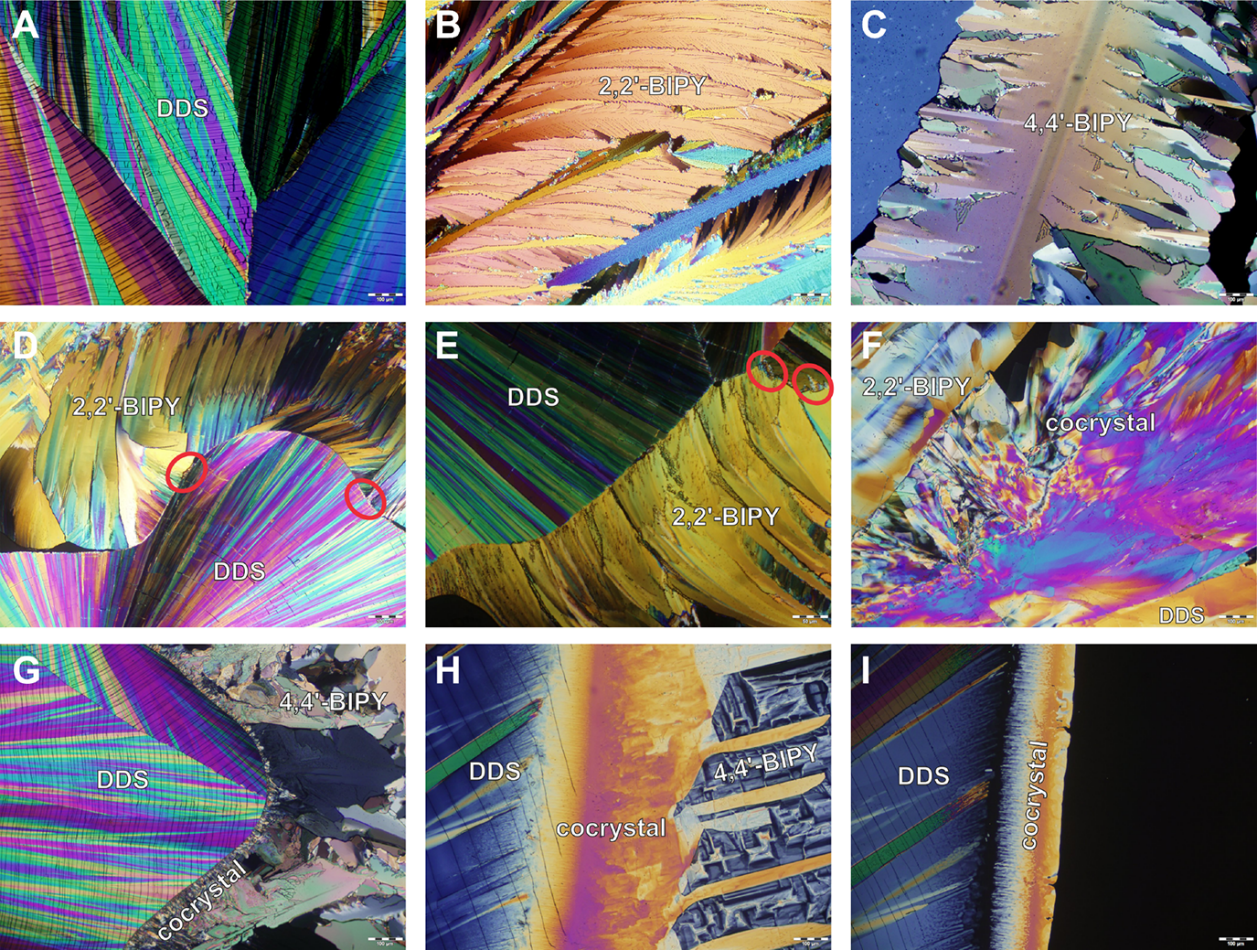
Hot-stage microscopy images (melt-film preparations) of (A) DDS, (B) 2,2′-BIPY, (C) 4,4′-BIPY, (D–F) contact preparation of DDS:2,2′-BIPY, and (G–I) contact preparation of DDS:4,4′-BIPY. Cocrystal formation is highlighted with a red circle in panels D and E. Scale bar corresponds to 100 μm.

The contact preparations of DDS:2,2′-BIPY and DDS:4,4′-BIPY clearly indicated the formation of cocrystals (Figure 7). However, the high melting point differences between DDS (form II: 178 °C) and the coformers (2,2′-BIPY: 70 °C and 4,4′-BIPY: 112 °C), as well as the high volatility of the BIPYs, posed challenges during the experiments.

Figure 7A–C illustrates the melt film preparations of DDS and BIPYs, while panels D–I show the contact preparations that were produced as described in Section 2.3. We observed small amounts of cocrystals at the contact zone (panels D and E: encircled in red, panels F–I: labeled), which could be enlarged by melting the cocrystal and BIPY (heating) and subsequent cooling to RT. The melting points of DDS:2,2′-BIPY and DDS:4,4′-BIPY were determined to be 99 and 174 °C, respectively, i.e., CC_22_-A (see next section) and CC_44_-B (2:1) had formed.

Both, liquid-assisted and dry grinding experiments of DDS and 2,2′-BIPY were performed, which resulted in two different cocrystal forms. It should be noted that CC_22_-B was never obtained phase pure in the grinding experiments (Table 3), and traces of the educts were always present. Increasing the duration of the grinding experiments beyond 1 h lead to an increase in the amount of DDS, which may be related to the volatility of 2,2′-BIPY.

**Table 3:** Results of the Mechanochemical Formation of DDS:2,2′-BIPY and DDS:4,4′-BIPY Cocrystals

	DDS:2,2′-BIPY			DDS:4,4′BIPY		
solvent	1:1	1:2	2:1	1:1	1:2	2:1
*t*-BuOH	CC_22_-B^a^	CC_22_-B + 22BIPY	CC_22_-B + DDS	CC_44_-A	CC_44_-A + 44BIPY	CC_44_-B
*i*-BuOAc	CC_22_-B^a^	CC_22_-B + 22BIPY	CC_22_-B + DDS	CC_44_-A	CC_44_-A + 44BIPY	CC_44_-B
DIPE	CC_22_-A + CC_22_-B	CC_22_-B + 22BIPY	CC_22_-A + CC_22_-B + DDS	CC_44_-A	CC_44_-A + 44BIPY	CC_44_-B
water	CC_22_-A	CC_22_-B + 22BIPY	CC_22_-A + DDS	CC_44_-B + 44BIPY	CC_44_-B + 44BIPY	CC_44_-B
*n*-heptane	CC_22_-A + CC_22_-B	CC_22_-B + 22BIPY	CC_22_-A + DDS	CC_44_-A	CC_44_-A + 44BIPY	CC_44_-B
dry	CC_22_-A	CC_22_-A + 22BIPY	CC_22_-A + DDS	CC_44_-A	CC_44_-A + 44BIPY	CC_44_-B

a Not phase pure (traces of educts detectable).

In contrast, CC_22_-A could be prepared phase pure in dry and water-assisted grinding experiments starting from a 1:1 molar ratio (Table 3). Based on the results, it could be concluded that DDS and 2,2′-BIPY form cocrystal polymorphs (1:1). CC_22_-A often formed first, but the longer the grinding time (LAG), the more CC_22_-B was obtained, indicating that CC_22_-B is the stable polymorph at (and slightly above) RT. Furthermore, we observed that, in dry grinding experiments, no conversion to CC_22_-B was seen within 1 h, which can be related to the absence of solvent, which accelerated the transformation in the other experiments.

Grinding experiments of DDS and 4,4′-BIPY (1:1) were performed in various solvents, and except for water, CC_44_-A was obtained. When water was used, a mixture of 4,4′-BIPY and CC_44_-B was obtained. CC_44_-B corresponds to the 2:1 cocrystal reported by Martins et al.,^47^ while CC_44_-A may correspond to the second cocrystal mentioned by the same authors, but no further characterization was provided. A starting ratio of 2:1 consistently lead to CC_44_-B, regardless of the solvent used. Thus, with the exception of water, the molar ratio of the educts defines, which of the two cocrystals is obtained. More detailed results of the mechanochemical experiments can be found in Tables S9 and S11 of the Supporting Information.

Slurry experiments were performed in DIPE, water, and *n*-heptane, chosen based on the results of the mechanochemical experiments. Saturated solutions (RT) of DDS and BIPYs in the respective solvents were prepared beforehand to overcome the solubility differences between them. In case of 2,2′-BIPY, a clear solvent trend was observed after one week. DIPE and *n*-heptane favored the formation of CC_22_-B, while the polar solvent water favored, similar to the grinding experiments, the formation of CC_22_-A (Table 4). Interestingly, in all of the DDS:2,2′-BIPY slurry experiments, CC_22_-A formed initially, but transformed to CC_22_-B in DIPE and *n*-heptane (for more details, see Table S10, Supporting Information). Nevertheless, obtaining phase pure CC_22_-B was problematic, likely due to slow conversion rates and solvent evaporation.

**Table 4:** Results of the DDS and BIPY Slurry Experiments in (Organic) Solvents

	DDS:2,2′-BIPY			DDS:4,4′-BIPY		
solvent	1:1	1:2	2:1	1:1	1:2	2:1
DIPE	CC_22_-B + 22BIPY	CC_22_-B + 22BIPY	CC_22_-B + 22BIPY^a^	CC_44_-A	CC_44_-A + 44BIPY	CC_44_-B + DDS^a^
water	CC_22_-A	CC_22_-A + 22BIPY	CC_22_-A + DDS	CC_44_-B + 44BIPY	CC_44_-B + 44BIPY	CC_44_-B
*n*-heptane	CC_22_-B + 22BIPY^a^*	CC_22_-A^a^ + CC_22_-B + 22BIPY	CC_22_-A+ CC_22_-B + DDS	CC_44_-A	CC_44_-A + 44BIPY	CC_44_-A + CC_44_-B + DDS

a Minor component.

The slurry experiments conducted with DDS:4,4′-BIPY in water produced CC_44_-B (along with educt excess) regardless of the starting ratio used (1:1, 1:2, or 2:1). When DIPE or *n*-heptane was used with a 1:1 or 1:2 molar ratio, CC_44_-A (plus excess 4,4′-BIPY) was formed. When a 2:1 molar mixture and DIPE or *n*-heptane were used, CC_44_-A formed initially and then slowly transformed into CC_44_-B. For further details, refer to Table S12 in the Supporting Information.

### 3.4 Dapsone:Bipyridine Cocrystals

#### 3.4.1 Structural Characterization of the DDS:2,2′-BIPY Cocrystals

**Figure 8:**
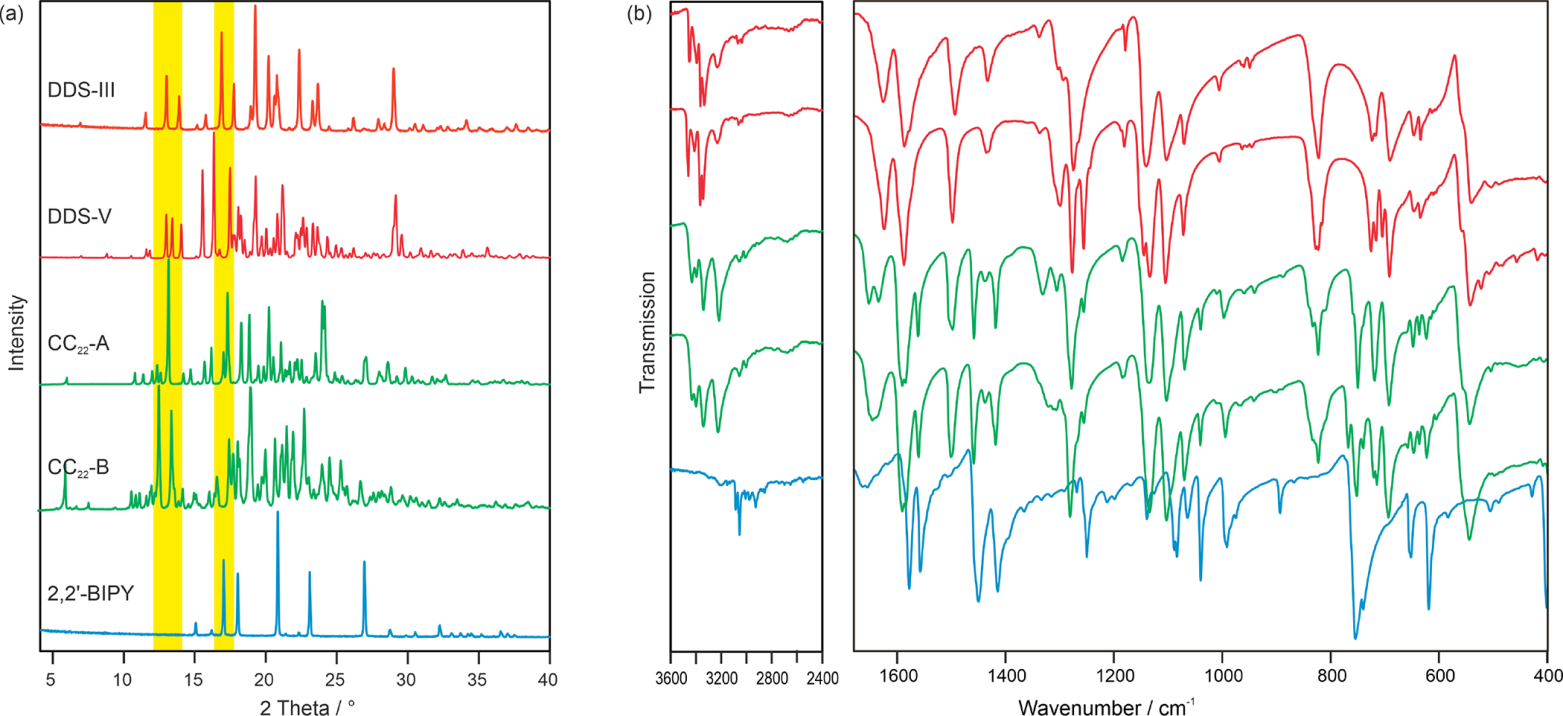
(a) PXRD and (b) IR spectra of DDS (forms III and V), DDS:2,2′-BIPY cocrystals (CC_22_-A and CC_22_-B), and 2,2′-BIPY. Key areas for differentiating the solid-state forms are highlighted in panel a.

Through the experimental screen, the first cocrystals of DDS and 2,2′-BIPY, namely, two 1:1 polymorphs, were obtained. Both cocrystals could be prepared using either water or organic solvents in grinding or slurry experiments, and the outcome was dependent on the solvent choice and stirring time. In the case of CC_22_-B, additional purification was necessary to eliminate any remaining 2,2′-BIPY (see Section 2.3). The PXRD patterns of the two cocrystals (Figure 8a) are distinguishable from the educts and from each other.

The IR spectra of the cocrystals were compared to those of the educts (Figure 8b). The valence and deformation vibrations of the DDS–NH_2_ groups can be seen in the range of 3340–3450 cm^–1^ and at approx. 1630 cm^–1^,^91^ respectively. The latter band is shifted and split into a doublet for CC_22_-A and into ≥3 bands for CC_22_-B, indicating a different crystal environment and potentially a higher Z′ structure. The SO_2_ deformation vibrations at 540–550 cm^–1^ are characteristic bands^91^ for DDS and the cocrystals. Valence vibrations of the pyridine rings can be observed at approx. 1415 and 1450 cm^–1^ in 2,2′-BIPY and the cocrystals. The spectra of the cocrystals exhibit characteristic bands for both DDS and 2,2′-BIPY and are different from the sum of the educts, thereby confirming cocrystal formation.

**Figure 9:**
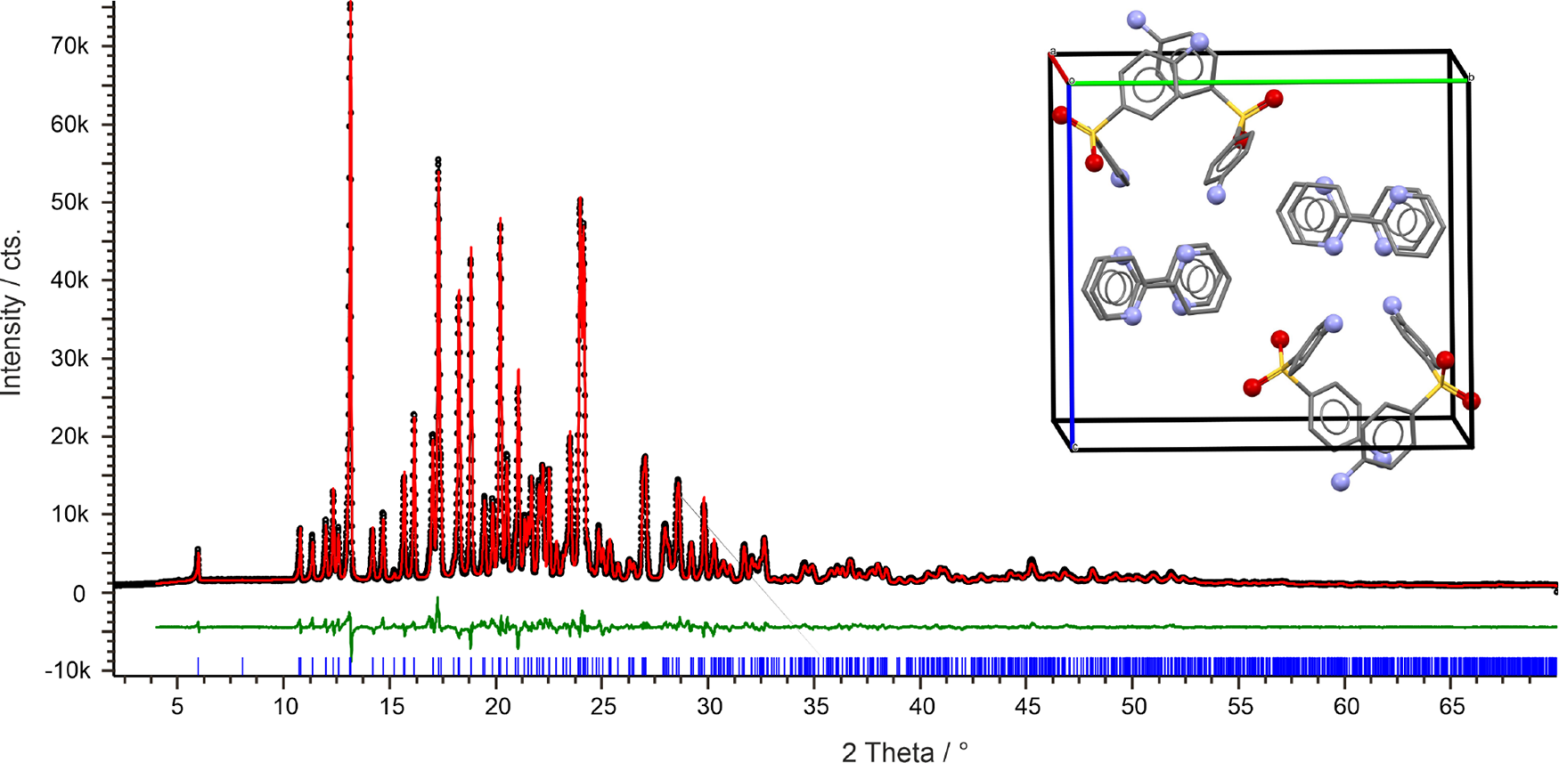
Powder X-ray diffraction pattern and Rietveld fit (rigid body) of CC_22_-A at 25 °C: observed (black points), calculated (red line), and difference profiles (green). Blue tick marks denote the peak positions. Inset shows the unit cell of CC_22_-A.

The PXRD pattern of CC_22_-A was successfully indexed (Figure 9). CC_22_-A crystallizes in the monoclinic *P*2_1_ symmetry with two DDS and two 2,2′-BIPY molecules in the asymmetric unit, confirming the 1:1 stoichiometry. Simulating annealing resulted in more than one structure that differed only in the orientation of the 2,2′-BIPY molecules, matching the predicted structures 22-1, 22-2, and 22-4. Rigid-body Rietveld refinements were performed using the three structures and resulted in similar fits, owing to the fact that the structures differ only in the positions of C(−H) and N atoms. Similarly, restrained Rietveld refinements or disorder modeling did not quantitatively change the results. Therefore, fixed-cell PBE-MBD* optimizations (lattice parameters fixed, atomic positions optimized) were performed, and the lowest energy structure was used for the final Rietveld refinement (with foreshortened H positions). It should be noted that full structure minimizations (lattice parameters and atomic positions optimized) did not alter the stability order of the potential structures, suggesting that CC-1 (the lowest energy structure on Figure 4d) corresponds to CC_22_-A.

In CC_22_-A, the DDS molecules adopt the characteristic bent conformation of the compound (C–S–C angle of 107–108°), and the BIPY molecules adopt the planar lowest energy conformation. Each of the DDS–NH_2_ groups forms two H-bonding interactions. Three of the −NH_2_ groups interact with DDS and BIPY, and the fourth −NH_2_ group interacts with DDS only. Each DDS oxygen atom serves as an H-bond acceptor. In case of 2,2′-BIPY, only three of the four possible acceptor sites form H-bonding interactions. Furthermore, aromatic interactions stabilize the cocrystal structure.

Table 5 and Figure S3 (Supporting Information) provide information on the strengths of the pairwise intermolecular interactions^82^ seen in CC_22_-A. Surprisingly, the strongest intermolecular interaction does not arise from H-bonding, but from a pair of DDS molecules interacting through aromatic (π···π and C–H···π) contacts (see the inset in Figure 9), accounting for −42.4 kJ mol^–1^ in pairwise energy. The N–H···Ο and N–H···N_BIPY_ interactions were calculated in the range of −38.5 to −28.0 kJ mol^–1^, and the remaining N–H···Ν_DDS_ interaction as −20.6 kJ mol^–1^. Other strong interactions arise from the 2,2′-BIPY molecule stacks along the *a* crystallographic axis (−24.6 and −24.7 kJ mol^–1^). In contrast, the strongest interaction seen in DDS (III and V) and 2,2′-BIPY were calculated as −43.1 and −18.6 kJ mol^–1^, respectively. These results suggest that a combination of multiple intermolecular interactions, including H-bonding and aromatic interactions, contributes to the stability of CC_22_-A.

**Table 5:** Strongest Pairwise Intermolecular Interactions^a^ Seen in CC_22_-A

		Å	kJ mol^–1^				
no.	interaction	*R*	*E*_E_	*E*_P_	*E*_R_	*E*_D_	*E*_tot_
1	DDS···DDS	3.85	–8.6	–3.8	–67.0	45.1	–42.4
2	N–H···N_BIPY_	7.89	–51.6	–12.1	–24.1	74.5	–38.5
3	N–H···O	9.41	–42.9	–9.6	–9.2	37.1	–37.5
4	N–H···N_BIPY_	8.08	–49.2	–11.8	–23.3	70.5	–37.5
5	N–H···N_BIPY_	8.17	–43.9	–9.7	–23.9	59.8	–37.4
6	N–H···O	9.67	–39.0	–8.4	–7.4	32.1	–34.1
7	N–H···O	8.20	–25.4	–7.5	–16.9	25.1	–31.6
8	N–H···O	8.20	–28.9	–11.2	–18.5	43.6	–28.0
9	BIPY··· BIPY	4.15	–1.8	–1.6	–43.2	25.8	–24.7
10	BIPY··· BIPY	4.09	–2.7	–1.7	–45.0	30.1	–24.6
11	DDS···DDS	8.79	–12.1	–2.3	–17.1	12.8	–21.5
12	N–H···N_DDS_	9.32	–18.2	–4.5	–13.3	22.1	–20.6

a Electrostatic (*E*_E_), polarization (*E*_P_), dispersion (*E*_D_), and exchange-repulsion (*E*_R_). *E*_tot_ = *k*_E_*E*_E_ + *k*_P_*E*_P_ + *k*_D_*E*_D_ + *k*_R_*E*_R_, with *k* being scale factors.^81^

We were unable to grow single crystals or solve the structure of CC_22_-B from PXRD. Although indexing resulted in potential triclinic *Z*′ = 4 structures, the preliminary simulation annealing results were unsatisfactory.

#### 3.4.2 Structural Characterization of the DDS:4,4′-BIPY Cocrystals

**Figure 10:**
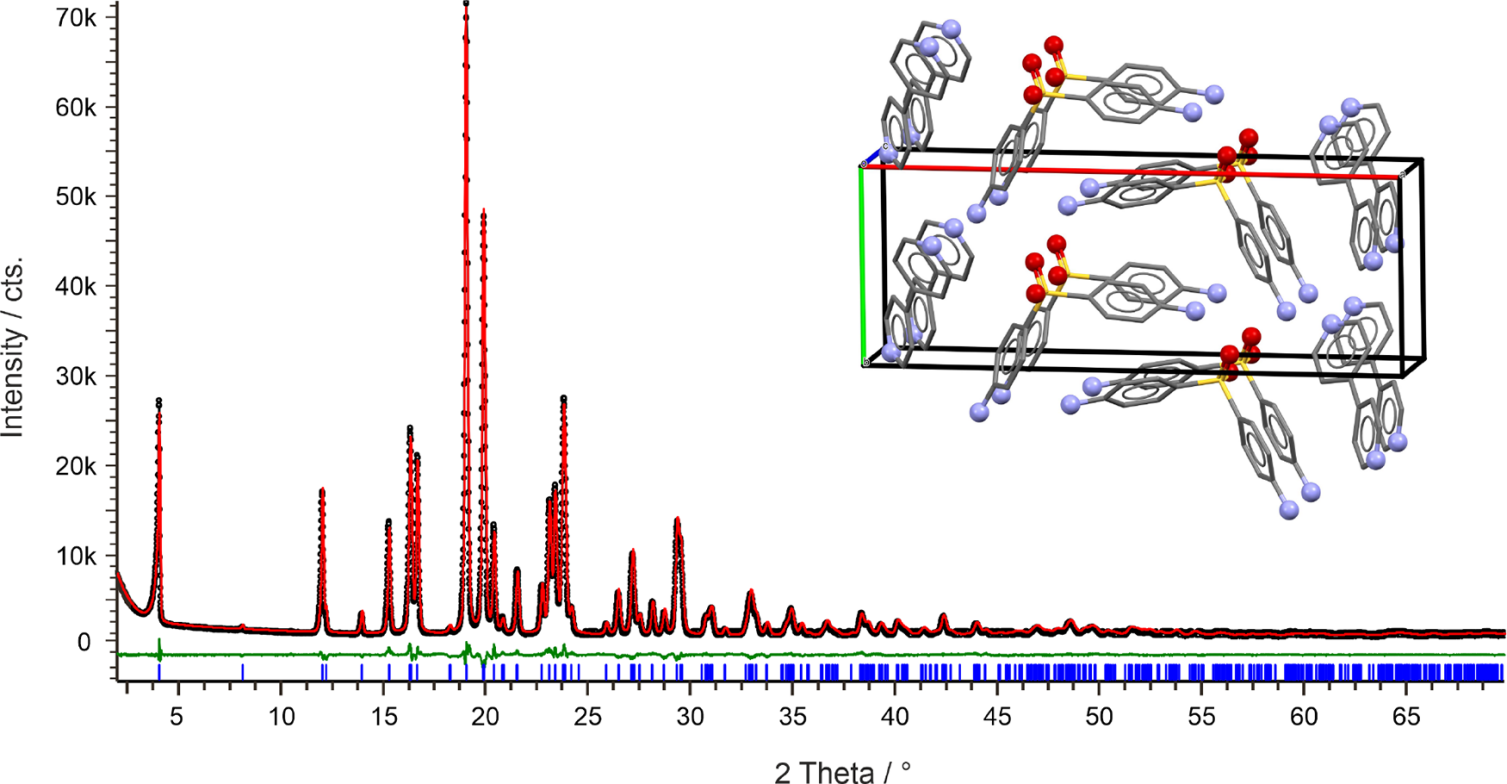
Powder X-ray diffraction pattern and Rietveld fit (rigid body) of CC_44_-A at 25 °C: observed (black points), calculated (red line), and difference profiles (green). Blue tick marks denote the peak positions. Inset shows the packing diagram of CC_44_-A.

The single-crystal structure of CC_44_-B has already been reported.^47^ In LAG and slurry experiments, phase pure cocrystal CC_44_-A was obtained. The 1:1 cocrystal crystallizes in the monoclinic space group *P*2_1_ with one DDS and one 4,4′-BIPY molecule in the asymmetric unit (Figure 10). The BIPY conformation closely resembles to the global minimum conformer,^62^ with a bipyridyl dihedral angle of 35°. One of the dapsone −NH2 groups forms hydrogen bonds (N–H···O) with symmetry-related DDS molecules, while the second −NH2 group interacts with another DDS molecule (N–H···O) and the coformer (N–H···N_BIPY_). Therefore, one of the −SO_2_ oxygen atoms acts as a double acceptor group. The packing comparison with the computationally generated structures (Figure 3e) revealed that the experimental structure (CC_44_-A) corresponds to the global minimum structure (44-1, Figure 6b).

**Figure 11:**
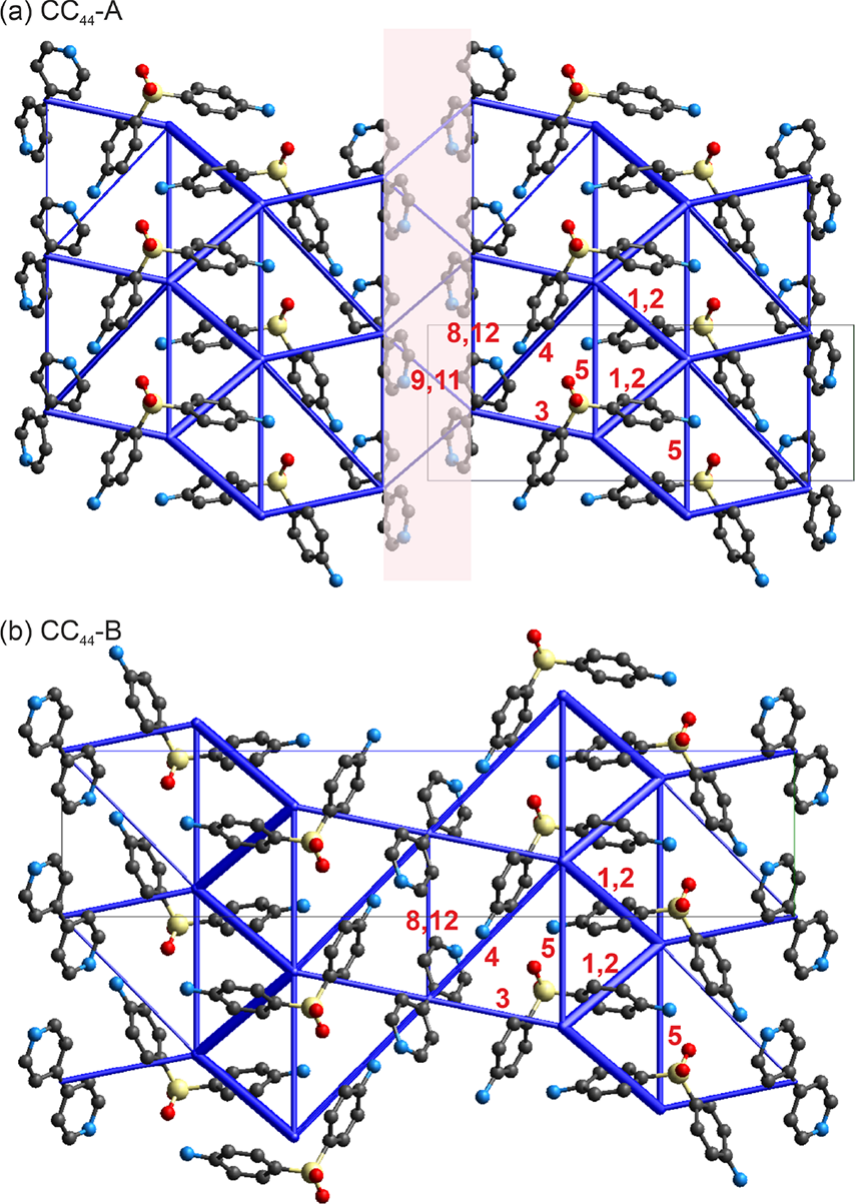
Energy framework diagram (total energy) for the (a) CC_44_-A and (b) CC_44_-B cocrystals. The energy scale factor is 60. Stabilizing contacts are shown in blue, and the thickness corresponds to the strength. Pairwise interaction energies <5 kJ mol^–1^ are omitted. Numbers correspond to the interactions listed in Table 6.

To quantify the interactions observed in the closely related DDS:4,4′-BIPY cocrystals (as described in Section 3.2), pairwise intermolecular energy calculations were performed. The resulting energy framework diagrams (Figure 11) provide an overview of the cocrystal intermolecular interactions and show a high degree of resemblance. Within the distance range of 3.8 Å of either DDS or 4,4′-BIPY, 15 or 13 pairwise contacts are observed for CC_44_-A and CC_44_-B, respectively, with an overlap of 13 interactions between the 2 cocrystals (Table 6). The strongest interactions result from the N–H···O H-bonds and account for −40.4 to −46.0 kJ mol^–1^ in pairwise energy (marked with 1 and 2 on Figure 11). The strongest interactions between DDS and 4,4′-BIPY were calculated as −23.3 to −29.9 kJ mol^–1^ and arise from N–H···N and C–H···π interactions, which are comparable in strengths (3, 4). DDS stacks along the corresponding *b* axes also significantly contribute to the strength of the crystals (5). Interactions unique to CC_44_-A (resulting from an additional 4,4′-BIPY molecule) account for −12.5 and −10.6 kJ mol^–1^ (9, 11). A transformation from the CC_44_-A to CC_44_-B would require breaking the interactions 9 and 11, as well as a 180° flip of every alternate double stack of DDS molecules.

**Table 6:** Pairwise Intermolecular Interactions^a^ Seen in CC_44_-A and CC_44_-B

interaction		CC_44_-A						CC_44_-B					
		Å	kJ mol^–1^					Å	kJ mol^–1^				
no.	type	*R*	*E*_E_	*E*_P_	*E*_R_	*E*_D_	*E*_tot_	R	*E*_E_	*E*_P_	*E*_R_	*E*_D_	*E*_tot_
1	NH···O	6.83	–30.5	–7.2	–36.7	39.4	–45.2	6.86	–31.1	–7.2	–36.9	39.4	–46.0
2	N–H···O	7.00	–20.6	–7.5	–36.6	30.5	–40.3	6.91	–20.6	–7.6	–36.7	30.5	–40.5
3	C–H···π	5.92	–16.3	–5.1	–39.2	40.7	–29.9	6.07	–8.6	–4.6	–34.1	30.1	–23.6
4	N–H···N	10.7	–41.8	–10.3	–10.9	54.3	–27.7	10.9	–39.2	–9.9	–10.0	48.3	–27.6
5	DDS···DDS	7.73	–17.1	–7.4	–12.5	15.9	–24.6	7.65	–16.5	–7.7	–13.3	17.3	–24.0
6	BIPY···BIPY	5.73	–5.0	–1.1	–26.1	17.0	–18.3	5.82	–1.3	–0.8	–20.1	6.4	–15.5
7	DDS···DDS	5.73	0.1	–6.6	–32.5	26.7	–16.7	5.82	–0.2	–6.4	–31.5	27.5	–15.4
8	BIPY···BIPY	9.62	–12.7	–2.9	–8.8	16.0	–13.4	9.61	–8.7	–1.5	–8.7	7.8	–13.1
9	BIPY···BIPY	5.92	–2.6	–0.8	–19.7	13.0	–12.5						
10	DDS···BIPY	9.02	–1.0	–2.3	–17.8	12.0	–10.9	9.08	–2.4	–1.9	–15.9	11.2	–11.0
11	BIPY···BIPY	8.05	–5.0	–2.3	–12.9	12.3	–10.6						
12	BIPY···BIPY	7.73	–3.5	–0.7	–10.8	7.3	–9.1	7.65	–2.3	–0.7	–9.4	4.6	–8.3
14	DDS···BIPY	11.54	1.3	–0.4	–1.9	0.2	–0.5	11.74	–0.2	–0.3	–1.4	0.0	–1.7
15	DDS···BIPY	8.26	4.9	–1.1	–8.2	4.1	–0.3	8.56	5.0	–1.2	–9.0	6.4	0.5

a Electrostatic (*E*_E_), polarization (*E*_P_), dispersion (*E*_D_), and exchange-repulsion (*E*_R_). *E*_tot_ = *k*_E_*E*_E_ + *k*_P_*E*_P_ + *k*_D_*E*_D_ + *k*_R_*E*_R_, with *k* being scale factors.^81^

#### 3.4.3 Thermal Analysis and Thermodynamic Stability

**Figure 12:**
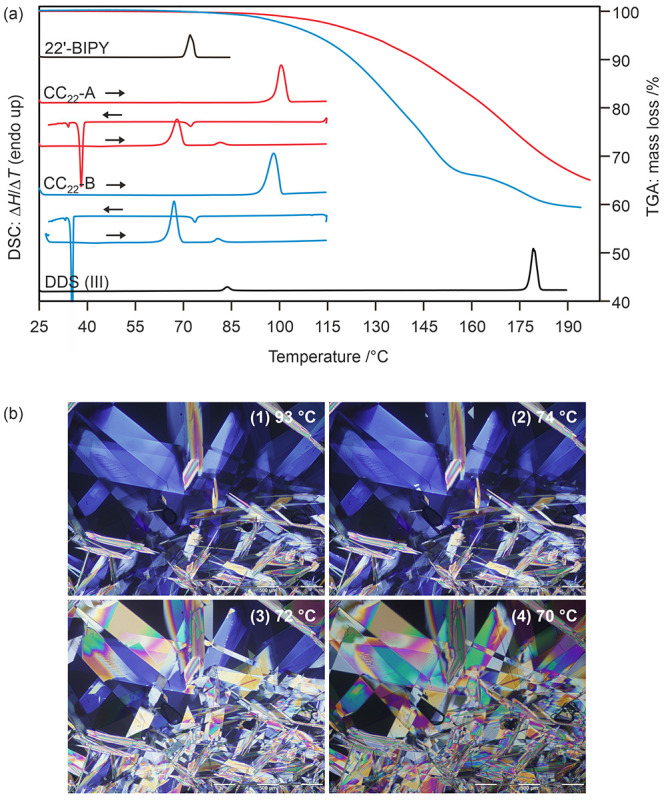
(a) TGA and DSC thermograms of 2,2′-BIPY, DDS, and the DDS:2,2′-BIPY cocrystals (CC_22_-A in red and CC_22_-B in blue). Heating rates of 10 °C min^–1^ were applied for all measurements. (b) The hot-stage microscopic investigation of CC_22_-A revealed that the melting of the sample resulted in the formation of dapsone crystals, which underwent a transformation from form II to form III upon cooling.

We conducted DSC and TGA experiments on both the phase-pure cocrystals and their starting materials to examine their thermal behavior. The melting points of 2,2′-BIPY and DDS form II are 70.3 ± 0.3 and 177.2 ± 0.1 °C, respectively (Figure 12a). The endotherm observed at approximately 82 °C in the dapsone DSC heating curve corresponds to the endothermic transformation from form III to form II.^38^ CC_22_-A and CC_22_-B show a single endothermic event upon heating, which corresponds to the melting of the cocrystals. CC_22_-A melts at 98.4 ± 0.2 °C, and CC_22_-B melts at 94.6 ± 0.3 °C. The measured Δ*H* values at the melting temperatures are 26.4 ± 0.3 and 27.9 ± 0.3 kJ mol^–1^ for cocrystals A and B, respectively. Recrystallization of DDS form II occurs rapidly upon melting the cocrystals, as demonstrated in Figure 12b for CC_22_-A. The DSC cooling and reheating curves of both samples are identical, and the characteristic phase transformation of DDS form II to form III can be seen at 72–74 °C upon cooling to RT, indicated by the small exotherm in the DSC curves and the change in birefringence in HSM investigations (cross-polarized light). Recrystallization of 2,2′-BIPY occurs at temperatures below 40 °C, as evidenced by the big exotherm upon cooling. The presence of 2,2′-BIPY and not cocrystal was confirmed with PXRD. The second heating curve of the cocrystals displays the eutectic between DDS and 2,2′-BIPY at 65 °C and the DDS form III to form II transformation. The measured II → III transformation enthalpy values (recorded on cooling) indicate that at least 90% of DDS recrystallized during the melting process of the cocrystals in all experiments.

The TGA curves for the two cocrystals (Figure 12a) exhibit significant mass loss due to the sublimation of the coformer. When subjected to slower heating rates, complete loss of 2,2′-BIPY was observed.

The relationship between CC_22_-A and CC_22_-B can be derived from the thermal data. CC_22_-A has a higher melting temperature but a lower heat of fusion value than CC_22_-B. Note that the enthalpy values provided are not just the heat of fusions, but a combination of fusion of the cocrystals and recrystallization of dapsone form II. Despite this, it can be concluded that CC_22_-A and CC_22_-B are enantiotropically related since similar quantities of DDS form II recrystallized. Slurry experiments in organic solvents indicate that CC_22_-B is the stable cocrystal polymorph at RT. CC_22_-A exhibits a very high kinetic stability as no transformation of CC_22_-A to CC_22_-B was observed at RT within two years (end of investigation time).

**Figure 13:**
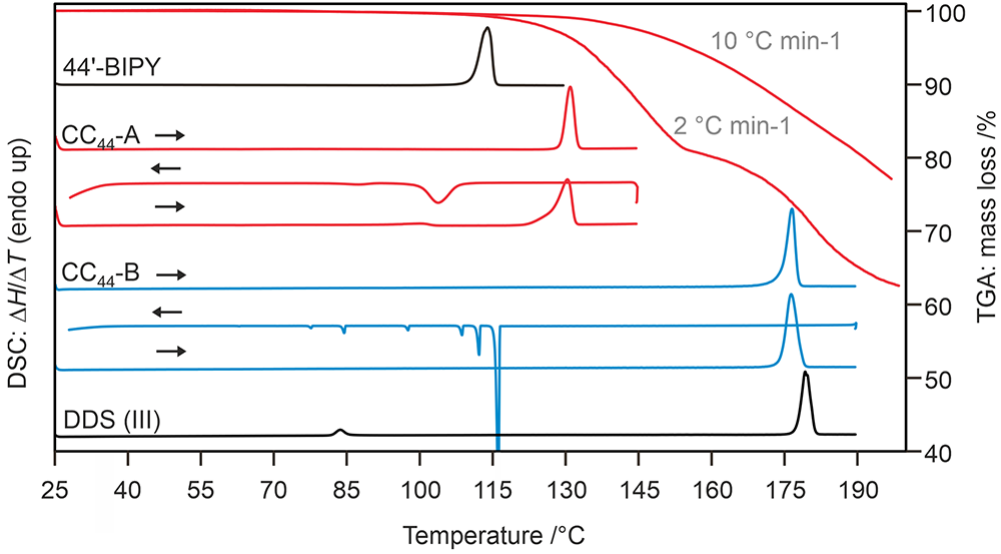
TGA and DSC thermograms of 4,4′-BIPY, DDS, and DDS:4,4′-BIPY cocrystals (CC_44_-A in red and CC_44_-B in blue). Heating rates of 10 °C min^–1^ were applied for all DSC measurements.

The results of the DSC investigations of CC_44_-A (1:1) and CC_44_-B (2:1) are presented in Figure 13, revealing melting points at 129.0 ± 0.2 and 174.4 ± 0.1 °C, respectively. When the melts of CC_44_-A and CC_44_-B were cooled, recrystallization of the respective cocrystals occurred at temperatures below 110 and 120 °C, respectively. Thus, the molar ratio determines the outcome of the melt crystallization product, assuming that no 4,4′-BIPY is lost during the experiment.

Martins et al.^47^ reported that CC_44_-B undergoes a phase transition between 118 and 150 °C, which apparently corresponded to the collapse of the cocrystal and reorganization into DDS. Our TGA experiments, combined with *ex situ* PXRD measurements, revealed that heating CC_44_-A above its melting point temperature (>129 °C) lead to the formation of CC_44_-B, with 0.5 moles of 4,4′-BIPY lost. Storing CC_44_-B at higher temperatures resulted in the formation of DDS form II, as previously described. The TGA curve measured at a slower heating rate (2 °C min^–1^) clearly shows the two-step loss of 4,4′-BIPY (Figure 13).

Due to the different stoichiometric ratios of the two 4,4′-BIPY cocrystals, it was not possible to determine the thermodynamic stability order of the cocrystals using DSC experiments alone. Therefore, lattice energy calculations were used to gain a better understanding of their stability. The cocrystal formation enthalpies, ΔΔ*E*_*F*_^CC^ (eq 5), were calculated for CC_44_-A and CC_44_-B, and resulted in −14.0 and −19.5 kJ mol^–1^, respectively. CC_44_-B was found to be more stable, possibly due to both 4,4′-BIPY N atoms being involved in H-bonding interactions, compared to CC_44_-A where only one N atom forms a strong intermolecular interaction (Table 6). No transformation of CC_44_-A to CC_44_-B was observed at RT during a two-year investigation period, likely due to the substantial structural rearrangements required (i.e., a 180° flip of every alternate common fragment, Figure 6a,b), which only occurred at higher temperatures during loss of BIPY.

Comparing the ΔΔ*E*_*F*_^CC^ values between CC_22_-A and CC_44_-A, it was found that the 2,2′-BIPY cocrystal (−11.6 kJ mol^–1^) gains less in stabilization energy than the 4,4′-BIPY cocrystals. As a result, CC_22_-A is calculated to be enthalpically less stable than CC_44_-A and CC_44_-B.

## 4 Conclusions

A combination of experimental and theoretical methods has led to the discovery of new cocrystal forms of the anti-infective drug DDS. This includes the identification of DDS:2,2′-BIPY cocrystal polymorphs and two different DDS:4,4′-BIPY multicomponent forms. The virtual cocrystal screening tools, including multicomponent hydrogen-bond (MCHB) propensity, molecular complementarity (MC), and molecular electrostatic potential (MEP) maps, performed well overall. However, the MC had some limitations, and it is important to consider geometrical aspects, such as input conformation, when estimating the cocrystallization tendency. MEP maps offer the advantage of being an energy-based method, allowing for the estimation of potential interaction strengths.

Crystal structure prediction (CSP) is a more time-consuming and precarious computational screening method. However, it provides unique information about the 3D packing and stability of each hypothetical cocrystal structure. The cocrystal stability could be estimated by calculating the difference between the lattice energy of the cocrystal and the single components, allowing for a ranking of stability for both same and different stoichiometric ratios.

The experimental cocrystal screen was challenging because DDS and BIPY have substantially different solubilities in water/organic solvents and different melting points. This significantly affects solution and thermal-based (e.g., hot-melt extrusion) cocrystal screening methodologies, respectively, which has to be seen as a general challenge in cocrystal research.

Most cocrystal screens focus on finding a cocrystal but do not account for cocrystal polymorphism. As shown in this study, the cocrystal formed initially may not necessarily be the product obtained upon longer reaction times (grinding, slurry experiments), as a transformation to a more stable product can occur. The DDS:BIPY cocrystals are not unique in this regard.^92,93^

Different cocrystallization tendencies and stabilities of the cocrystals have been encountered, despite the compounds being related. Nevertheless, all four identified cocrystals exhibit high stability at room conditions. The position of the BIPY N atoms defines packing preferentiality. In the case of DDS:2,2′-BIPY, CSP studies revealed that the 2,2′-BIPY molecules can adopt different orientations in the same structure due to the planarity of the molecule. In the case of DDS:4,4′-BIPY, there is one energetically highly favored 2D packing arrangement, involving DDS and 4,4′-BIPY, which can be seen among 1:1 and 2:1 cocrystals. Thus, structure determination, in this study via PXRD data or CSP, enabled the molecular-level analysis of these materials and offered insight into their structure–property relationships.

## Supporting Information Available

Virtual cocrystal screening, computational generation of the single- and multicomponent lattice energy landscapes, experimental cocrystal screening, pairwise intermolecular energy calculations, and IR spectra (PDF)
