# Supplementary material for: Exploring the Supramolecular Interactions and Thermal Stability of Dapsone:Bipyridine Cocrystals by Combining Computational Chemistry with Experimentation
Source: Cryst Growth Des. 2023 May 3;23(6):4638–54. doi: 10.1021/acs.cgd.3c00387 (PMC10251420; doi:10.1021/acs.cgd.3c00387)
Supplement: Supplementary file 1 — cg3c00387_si_001.pdf [file cg3c00387_si_001.pdf]

Supplementary Information

Exploring the supramolecular interactions and thermal stability of dapsone and bipyridine cocrystals by combining computational chemistry with experimentation

Florian Racher, Tom L. Petrick and Doris E. Braun*

Institute of Pharmacy, University of Innsbruck, Innrain 52c, 6020 Innsbruck, Austria

*Email: doris.braun@uibk.ac.at

Table of Contents

1. Virtual cocrystal screening 3
1.1. Multi-component hydrogen-bond (MCHB) propensity screen for dapsone cocrystals 3
1.2. Molecular complementarity screen for dapsone cocrystals 7
1.3. Molecular electrostatic potential (MEP) maps 8
1.4. Computational generation of dapsone, 2,2'-bipyridine and 4,4'-bipyridie low-energy structures 9
1.5. Computational generation of dapsone:2,2'-bipyridine (1:1) low-energy structures 13
1.6. Computational generation of dapsone:4,4'-bipyridine (1:1) low-energy structures 14
2. Experimental Dapsone:2,2'-Bipyridine cocrystal screen 15
2.1. Grinding experiments (dry and liquid-assisted) 15
2.2. Slurry experiments in organic solvents 18
3. Experimental Dapsone:4,4'-Bipyridine cocrystal screen 19
3.1. Grinding experiments (dry and liquid-assisted) 19
3.2. Slurry experiments in organic solvents 22
4. Characterization of the cocrystals 23
References 25

1. Virtual cocrystal screening

1.1. Multi-component hydrogen-bond (MCHB) propensity screen for dapsone cocrystals

Table S1. Multi-component hydrogen-bond propensity screen results: D – dapsone and C – coformer. In green the 2,2'-bipyridine and 4,4'-bipyridine results.

Rank	Coformer (C)	Multi-component score	Max interaction	Max D:C or C:D propensity	Max D:D propensity	Max C:C propensity
1	*DL*-malic acid	0.34	D:C	0.93	0.59	0
2	3,5-dihydroxybenzoic acid	0.25	D:C	0.91	0.66	0.6
**3**	**4,4'-bipyridine**	**0.23**	**D:C**	**0.86**	**0.64**	**0**
4	catechol	0.23	D:C	0.9	0.67	0
5	succinic acid anhydride	0.23	D:C	0.92	0.69	0
6	pyrazine	0.22	D:C	0.86	0.64	0
7	3-methylpyridine	0.21	D:C	0.85	0.64	0
8	phthalic acid anhydride	0.16	D:C	0.85	0.69	0
9	alitame	0.15	D:C	0.8	0.64	0.66
10	orotic acid	0.15	D:C	0.82	0.67	0.61
11	*t*-butylamine	0.15	D:C	0.79	0.63	0.63
**12**	**2,2'-bipyridine**	**0.14**	**D:C**	**0.78**	**0.64**	**0**
13	camphor	0.14	D:C	0.79	0.65	0
14	4-acetamidobenzoic acid	0.13	D:C	0.85	0.71	0.72
15	nicotinic acid	0.13	D:C	0.84	0.71	0.64
16	caprolactam	0.12	D:C	0.94	0.71	0.82
17	xanthine	0.12	D:C	0.83	0.65	0.71
18	acetic acid	0.11	D:C	0.76	0.64	0.45
19	*L*-methionine	0.11	D:C	0.71	0.6	0.56
20	N-ethylacetamide	0.11	D:C	0.91	0.68	0.8
21	(-)-camphorsulfonic acid	0.1	D:C	0.84	0.59	0.74
22	2-oxoglutaric acid	0.1	D:C	0.75	0.65	0.52
23	cholic acid	0.1	D:C	0.75	0.65	0.62
24	oxalic acid	0.1	D:C	0.8	0.71	0.44
25	ketoglutaric acid	0.09	D:C	0.74	0.65	0.52
26	L-pyroglutamic acid	0.09	D:C	0.91	0.67	0.82
27	piperazine	0.09	D:C	0.74	0.65	0.61
28	caffeine	0.08	D:C	0.76	0.68	0
29	glutaric acid anhydride	0.08	D:C	0.74	0.67	0
30	*L*-lactic acid	0.08	D:C	0.73	0.65	0.55
31	lauric acid	0.08	D:C	0.73	0.65	0.57
32	stearic acid	0.08	D:C	0.73	0.65	0.58
33	azelaic acid	0.07	D:C	0.72	0.65	0.54
34	caprylic acid	0.07	D:C	0.72	0.65	0.56
35	*D*-glucuronic acid	0.07	D:C	0.74	0.67	0.46

Rank	Coformer (C)	Multi-component score	Max interaction	Max D:C or C:D propensity	Max D:D propensity	Max C:C propensity
36	*L*-leucine	0.07	D:C	0.67	0.6	0.5
37	lactobionic acid	0.07	D:C	0.73	0.66	0.63
38	sebacic acid	0.07	D:C	0.73	0.65	0.55
39	isonicotinamide	0.06	D:C	0.73	0.64	0.67
40	malonic acid	0.06	D:C	0.72	0.66	0.56
41	nicotinamide	0.06	D:C	0.72	0.64	0.66
42	suberic acid	0.06	D:C	0.72	0.66	0.54
43	adipic acid	0.05	D:C	0.71	0.65	0.52
44	glutaric acid	0.05	D:C	0.71	0.66	0.51
45	glycine	0.05	D:C	0.78	0.61	0.73
46	isovaleric acid	0.05	D:C	0.7	0.65	0.5
47	*L*-ascorbic acid	0.05	D:C	0.68	0.63	0.58
48	pimelic acid	0.05	D:C	0.71	0.66	0.53
49	succinic acid	0.05	D:C	0.71	0.66	0.52
50	mannitol	0.04	D:C	0.7	0.65	0.54
51	gluconic acid	0.03	D:C	0.67	0.64	0.5
52	glycolic acid	0.03	D:C	0.68	0.65	0.51
53	hippuric acid	0.03	D:C	0.72	0.69	0.44
54	*L*-tartaric acid	0.03	D:C	0.68	0.66	0.45
55	malic acid	0.03	D:C	0.69	0.65	0.46
56	sucrose	0.03	D:C	0.78	0.74	0.75
57	phthalamide	0.02	D:C	0.72	0.68	0.7
58	4-aminobenzoic acid	0.01	C:D	0.72	0.71	0.58
59	*L*-tryptophan	0.01	D:C	0.61	0.6	0.58
60	lactose	0.01	D:C	0.77	0.72	0.76
61	thymidine	0.01	D:C	0.7	0.69	0.56
62	acesulfame	0	D:C	0.65	0.65	0.59
63	*cis*-aconitic acid	0	D:C	0.68	0.68	0.52
64	galactraic acid	0	D:C	0.66	0.65	0.4
65	methylparaben	0	D:D	0.7	0.71	0.37
66	octyl gallate	0	D:D	0.67	0.68	0.65
67	pyridoxine	0	D:D	0.67	0.67	0.44
68	theophylline	0	D:D	0.67	0.68	0.38
69	thiourea	0	C:D	0.97	0.69	0.97
70	4-amino-2-hydroxybenzoic acid	-0.01	D:D	0.72	0.73	0.53
71	hydrocaffeic acid	-0.01	D:D	0.66	0.67	0.38
72	*L*-phenylalanine	-0.01	D:D	0.6	0.61	0.37
73	oxalic acid	-0.01	C:C	0.68	0.61	0.69
74	picolinic acid	-0.01	D:D	0.67	0.68	0.49

Rank	Coformer (C)	Multi-component score	Max interaction	Max D:C or C:D propensity	Max D:D propensity	Max C:C propensity
75	propyl gallate	-0.01	D:D	0.67	0.68	0.65
76	anthranilic acid	-0.02	D:D	0.69	0.71	0.52
77	ethylparaben	-0.02	D:D	0.68	0.7	0.35
78	flavone	-0.02	D:D	0.64	0.66	0
79	*L*-glutamine	-0.02	C:C	0.78	0.61	0.8
80	propyl paraben	-0.02	D:D	0.68	0.7	0.34
81	glutamic acid	-0.04	C:C	0.92	0.67	0.97
82	*RS*-phenylsuccinic acid	-0.04	D:D	0.63	0.67	0.35
83	*S*-phenylsuccinic acid	-0.04	D:D	0.62	0.66	0.35
84	vanillin	-0.04	D:D	0.69	0.72	0.32
85	(+)-camphoric acid	-0.05	D:D	0.61	0.65	0.29
86	citric acid	-0.05	D:D	0.62	0.66	0.32
87	γ-aminobutyric acid	-0.05	C:C	0.93	0.66	0.98
88	phenylacetic acid	-0.05	D:D	0.6	0.65	0.32
89	Acetylenedicarboxylic acid	-0.06	D:D	0.66	0.73	0.5
90	urea	-0.06	C:C	0.91	0.71	0.97
91	3-aminobenzoic acid	-0.07	C:C	0.91	0.59	0.99
92	fumaric acid	-0.08	D:D	0.64	0.72	0.45
93	maleic acid	-0.08	D:D	0.65	0.72	0.44
94	sorbic acid	-0.09	D:D	0.63	0.72	0.42
95	caffeic acid	-0.11	D:D	0.6	0.71	0.41
96	saccharin	-0.11	D:D	0.56	0.67	0.4
97	*t*-cinnamic acid	-0.12	D:D	0.59	0.72	0.4
98	vanillic acid	-0.14	D:D	0.56	0.7	0.35
99	benzoic acid	-0.15	D:D	0.56	0.71	0.38
100	isophthalic acid	-0.15	D:D	0.56	0.72	0.38
101	terephthalic acid	-0.15	D:D	0.57	0.72	0.4
102	trimesic acid	-0.16	D:D	0.56	0.72	0.38
103	phthalic acid	-0.17	D:D	0.54	0.71	0.34
104	4-hydroxybenzoic acid	-0.18	D:D	0.55	0.73	0.33
105	protocatechuic acid	-0.18	D:D	0.55	0.74	0.33
106	3-hydroxybenzoic acid	-0.19	D:D	0.54	0.73	0.32
107	β-resorcylic acid	-0.19	D:D	0.55	0.74	0.32
108	gallic acid	-0.19	D:D	0.55	0.74	0.33
109	gentisic acid	-0.19	D:D	0.54	0.74	0.31
110	γ-resorcylic acid	-0.2	D:D	0.54	0.74	0.3
111	salicylic acid	-0.2	D:D	0.54	0.73	0.31
112	pamoic acid	-0.22	D:D	0.52	0.74	0.28
113	hydroquinone	-0.24	D:D	0.48	0.72	0.19

Rank	Coformer (C)	Multi-component score	Max interaction	Max D:C or C:D propensity	Max D:D propensity	Max C:C propensity
114	resorcinol	-0.25	D:D	0.46	0.72	0.18
115	3-*t*-butyl-4-hydroxyanisole	-0.29	D:D	0.43	0.71	0.1
116	*t*-butylhydroxyanisole	-0.3	D:D	0.41	0.71	0.09

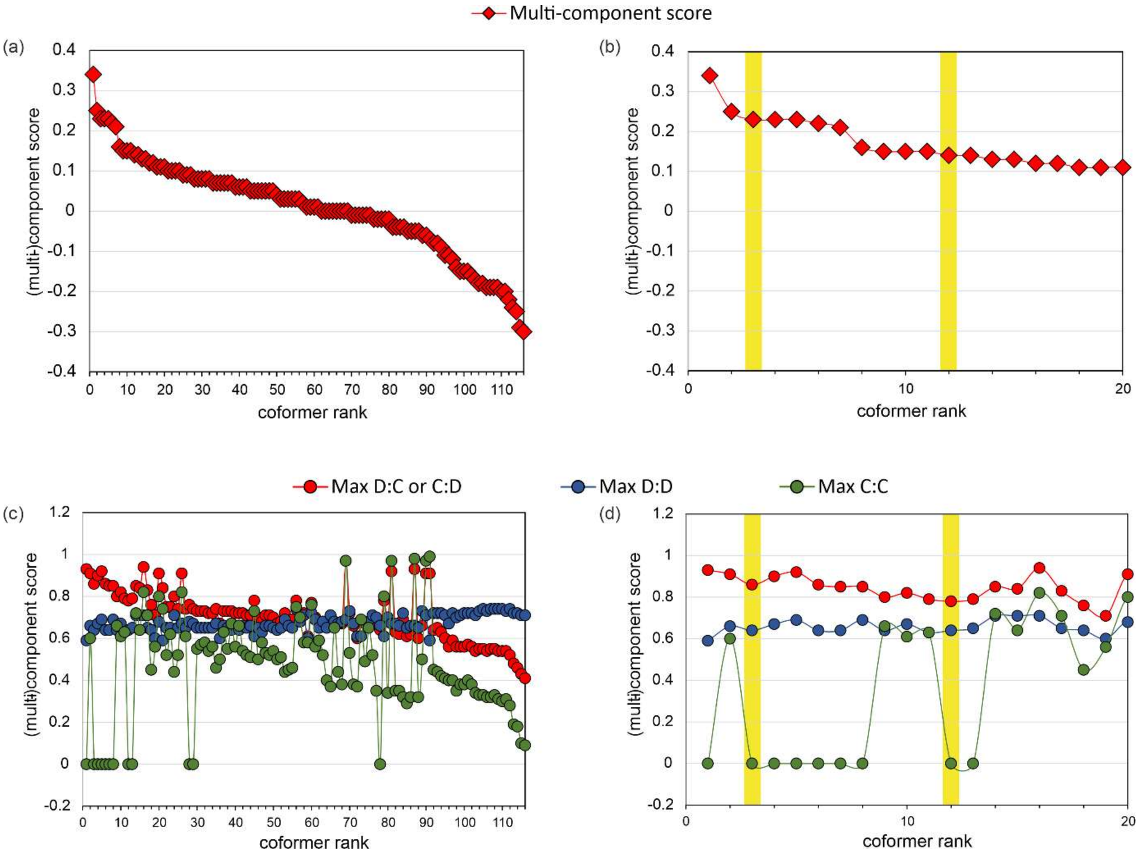

Figure S1. Multi-component hydrogen-bond propensity screen results: (a) and (b) multicomponent scores, (c) and (d) max. D:C (C:D), D:D and C:C propensity scores. In (b) and (d) the 4,4'-bipyridine (3) and 2,2'-bipyridine coformers (12) are highlighted.

1.2. Molecular complementarity screen for dapsone cocrystals

The results for the molecular complementarity search[1] of the top 20 ranked coformers (MCHB) are given in Table S2.

Table S2. Search for suitable coformers of dapsone via molecular complementarity (MC).

Rank (MCHB)	Coformer	**Overall**	**M/L axis ratio**	**M/L axis ratio**	**S axis (Å)**	**S axis (Å)**	**S/L axis ratio**	**S/L axis ratio**	**dipole moment magnitude (Debye)**	**dipole moment magnitude (Debye)**	**fraction of Nitrogen and Oxygen**	**fraction of Nitrogen and Oxygen**
1	*DL-malic* acid	FAIL	0.675	pass	5.581	pass	0.64	pass	2.691	pass	0.556	fail
2	3,5-dihydroxybenzoic acid	FAIL	0.988	fail	4.063	pass	0.463	pass	1.71	pass	0.364	pass
3	4,4'-bipyridine	PASS	0.644	pass	4.099	pass	0.401	pass	0.11	pass	0.167	pass
4	catechol	FAIL	0.901	pass	3.532	fail	0.512	pass	1.86	pass	0.25	pass
5	succinic acid anhydride	FAIL	0.731	pass	4.245	pass	0.569	pass	4.282	pass	0.429	fail
6	pyrazine	FAIL	0.904	pass	3.41	fail	0.525	pass	0	pass	0.333	pass
7	3-methylpyridine	PASS	0.814	pass	4.167	pass	0.506	pass	1.437	pass	0.143	pass
8	phthalic acid anhydride	FAIL	0.907	pass	3.473	fail	0.42	pass	4.027	pass	0.273	pass
9	alitame	PASS	0.656	pass	7.989	pass	0.61	pass	1.98	pass	0.318	pass
10	orotic_acid	FAIL	0.824	pass	3.44	fail	0.376	pass	1.447	pass	0.545	fail
11	*t-butylamine*	PASS	0.914	pass	5.954	pass	0.886	pass	0.771	pass	0.2	pass
12	2,2'-bipyridine	FAIL	0.584	pass	3.422	fail	0.296	fail	0	pass	0.167	pass
13	camphor	PASS	0.55	pass	7.722	pass	0.544	pass	3.812	pass	0.091	pass
14	4-acetamidobenzoic acid	FAIL	0.536	pass	4.174	pass	0.32	fail	1.612	pass	0.308	pass
15	nicotinic acid	FAIL	0.74	pass	3.457	fail	0.379	pass	1.652	pass	0.333	pass
16	caprolactam	PASS	0.95	pass	5.535	pass	0.696	pass	2.061	pass	0.25	pass
17	xanthine	FAIL	0.824	pass	3.405	fail	0.368	pass	1.202	pass	0.545	fail
18	acetic acid	FAIL	0.882	pass	4.175	pass	0.699	pass	0.86	pass	0.5	fail
19	*L-methionine*	PASS	0.681	pass	5.479	pass	0.533	pass	2.085	pass	0.333	pass
20	N-ethylacetamide	PASS	0.685	pass	4.169	pass	0.478	pass	1.656	pass	0.333	pass

1.3. Molecular electrostatic potential (MEP) maps

Table S3. Molecular electrostatic potential (MEP) maps: calculated energy gain (ΔE, in kJ mol^{-1}) for different DDS:BIPY stoichiometries.

Coformer/Stoichiometry	2:1	1:1	1:2
2,2'-BIPY	−7.49	−7.83	−8.08
4,4'-BIPY	−19.18	−17.90	−20.35

1.4. Computational generation of dapsone, 2,2’-bipyridine and 4,4’-bipyridie low-energy structures

The lattice energies of the previously published computationally generated low-energy structures of dapsone[2] and the bipyridines[3] were recalculated using the method described in the manuscript (generation of the cocrystal structures). The lists of the low-energy structures are given below. The lists of cocrystal structures are provided in the next sections.

Table S4. Computationally generated low-energy **dapsone** structures. The experimental structures are highlighted in green.

Str.	Space group	Cell parameters						E_{CASTEP}/	PI
		a/Å	*b*/Å	*c*/Å	*α*/°	*β*/°	*γ*/°	kJ mol^{-1}	
form V	$P2_1/n$	29.138	5.646	30.005	90	115.59	90	-368716.24	0.749
form III	$P2_12_12_1$	5.508	7.986	26.076	90	90	90	-368716.03	0.727
1311	$Pca2_1$	15.616	5.671	25.855	90	90	90	-368716.01	0.727
dehy	$C2/c$	48.553	11.382	12.917	90	92.75	90	-368714.53	0.699
21	$Pna2_1$	5.601	26.747	7.845	90	90	90	-368714.42	0.713
form I	$P2_1/c$	18.984	8.268	16.424	90	114.73	90	-368713.37	0.713
591	$Pca2_1$	15.480	5.834	25.708	90	90	90	-368713.21	0.717
form II	$P2_12_12_1$	5.707	7.886	25.057	90	90	90	-368712.08	0.747
155	$Pna2_1$	14.116	27.010	6.075	90	90	90	-368711.40	0.724
4478	$P2_1/c$	14.618	5.698	15.310	90	117.12	90	-368711.15	0.734
6694	$Pbca$	15.772	16.278	18.877	90	90	90	-368710.69	0.684
2871	$P2_1$	5.565	7.823	27.121	90	90.50	90	-368710.56	0.708
2291	$Pna2_1$	14.994	5.636	26.370	90	90	90	-368710.21	0.750
3410	$P2_1/c$	27.132	8.156	10.753	90	100.65	90	-368710.18	0.715
987	$P2_1/c$	27.302	5.690	15.031	90	104.26	90	-368709.93	0.738
1772	$P2_1/c$	26.776	8.161	10.788	90	100.69	90	-368709.44	0.722
409	$P2_1/c$	5.833	28.081	14.016	90	93.77	90	-368709.33	0.729
944	$Pbca$	5.769	15.197	26.649	90	90	90	-368709.08	0.712
69	$P2_1/c$	26.976	5.786	15.179	90	105.77	90	-368708.94	0.732
322	$P2_1$	5.647	7.974	25.667	90	90.75	90	-368708.37	0.726
5504	$Pca2_1$	29.248	5.722	13.903	90	90	90	-368708.31	0.718
DCM_desolv	$P2_1/n$	8.451	16.227	19.281	90	99.13	90	-368708.27	0.634
form IV	$Pca2_1$	16.130	8.233	17.465	90	90	90	-368708.01	0.721
180	$P2_1/c$	5.920	7.867	24.937	90	92.09	90	-368707.91	0.717
58	$Pna2_1$	15.347	5.959	25.533	90	90	90	-368707.84	0.717
61	$P2_1/c$	26.510	5.931	15.265	90	105.05	90	-368707.81	0.720
1389	$P2_1/c$	7.975	13.473	10.418	90	92.31	90	-368707.78	0.746
188	$P2_1/c$	5.927	29.479	13.192	90	96.24	90	-368707.73	0.729
500	$P2_12_12_1$	5.905	13.167	29.571	90	90	90	-368707.64	0.729
2423	$P2_1/n$	8.431	15.127	18.877	90	98.88	90	-368707.42	0.701
6150	$Pna2_1$	15.140	5.950	25.679	90	90	90	-368707.35	0.724
499	$P2_12_12_1$	5.902	13.177	29.461	90	90	90	-368707.19	0.724
724	$P2_1/c$	26.508	5.738	15.084	90	93.33	90	-368707.17	0.728
198	$Pca2_1$	5.788	26.654	14.954	90	90	90	-368707.03	0.722
1606	Ia	15.021	5.679	27.819	90	103.13	90	-368706.89	0.726
1063	$P2_1/n$	15.376	7.979	19.065	90	98.61	90	-368706.76	0.723

Str.	Space group	Cell parameters						E_{CASTEP}/ kJ mol^{-1}	PI
		a/Å	*b*/Å	*c*/Å	*α*/°	*β*/°	*γ*/°		
47	*Pca*2$_1$	7.923	11.873	24.144	90	90	90	-368706.62	0.738
33	*P*2$_1$/*c*	11.697	12.786	7.751	90	100.92	90	-368706.52	0.734
1297	*P*2$_1$/*c*	25.777	5.723	15.290	90	92.02	90	-368706.52	0.740
1	*P*2$_1$/*c*	11.697	12.764	7.747	90	100.73	90	-368706.49	0.736
196	*Ia*	15.011	5.828	27.402	90	104.16	90	-368706.46	0.722
15	*P*2$_1$/*c*	11.703	12.761	7.745	90	100.27	90	-368706.38	0.735
5243	*P*2$_1$	5.660	26.417	8.159	90	106.41	90	-368706.30	0.719

Table S5. Computationally generated low-energy **2,2'-bipyridine** structures. The experimental structure is highlighted in green.

Str.	Space group	Cell parameters						E_{CASTEP}/ kJ mol^{-1}	PI
		a/Å	*b*/Å	*c*/Å	*α*/°	*β*/°	*γ*/°		
204	*P*2$_1$/*n*	5.419	6.043	11.440	90	94.41	90	-217940.26	0.776
240	*P*2$_1$/*n*	6.529	11.342	10.454	90	100.78	90	-217938.38	0.762
877	*P*2$_1$/*n*	5.638	11.730	6.105	89.86	107.01	90.40	-217938.22	0.753
3011	*P*2$_1$2$_1$2$_1$	7.164	9.746	11.031	90	90	90	-217937.43	0.757
299	*Pbca*	5.860	12.637	20.618	90	90	90	-217936.53	0.761
966	*P*2$_1$/*n*	3.980	8.755	10.944	90	95.63	90	-217936.53	0.764
1759	*Pbca*	11.546	10.267	12.931	90	90	90	-217936.36	0.759
539	*P*2$_1$/*c*	10.512	5.920	12.836	90	103.76	90	-217936.29	0.748
503	*P*2$_1$/*n*	5.651	11.364	11.688	90	90.67	90	-217936.17	0.773
118	*P*2$_1$/*c*	3.871	9.213	10.792	90	100.14	90	-217935.81	0.767
1514	*Pca*2$_1$	11.034	8.919	7.819	90	90	90	-217935.80	0.756
699	*Aba*2	22.818	7.251	9.461	90	90	90	-217935.73	0.745
1805	*P*2$_1$2$_1$2$_1$	6.396	10.952	11.521	90	90	90	-217935.37	0.713
12	*Pna*2$_1$	14.523	4.744	10.994	90	90	90	-217935.08	0.769
1050	*P*2$_1$/*n*	5.491	11.793	11.731	90	97.57	90	-217934.80	0.770
244	*C*2/*c*	20.685	5.524	13.588	90	100.14	90	-217934.53	0.758
1132	*P*2$_1$/*n*	5.549	9.236	14.615	90	92.69	90	-217934.45	0.777
3751	*P*2$_1$/*n*	5.027	13.553	11.251	90	93.84	90	-217934.33	0.760
128	*P*2$_1$2$_1$2$_1$	5.026	11.417	13.088	90	90	90	-217934.17	0.773
654	*P*2$_1$/*n*	5.616	11.399	11.974	90	96.80	90	-217934.13	0.763
627	*P*2$_1$2$_1$2$_1$	5.151	10.379	14.256	90	90	90	-217934.12	0.764
419	*Pca*2$_1$	22.796	3.867	8.724	90	90	90	-217933.89	0.756
35	*Pna*2$_1$	16.981	3.990	11.162	90	90	90	-217933.73	0.756
1807	*C*2/*c*	11.194	8.263	16.926	89.95	106.39	89.46	-217933.31	0.773
253	*P*2$_1$/*n*	7.376	9.099	12.044	90	105.27	90	-217933.27	0.773
1269	*Pbca*	11.749	7.078	18.635	90	90	90	-217933.19	0.748
435	*I*2/*c*	11.088	11.372	13.114	90	114.37	90	-217933.11	0.770
570	*P*2$_1$2$_1$2$_1$	3.944	8.755	21.946	90	90	90	-217933.02	0.770
848	*P*-1	5.183	10.600	7.321	89.74	104.47	90.34	-217932.97	0.744
4527	*Pbca*	7.179	9.551	11.484	90	90	90	-217932.50	0.740
3081	*Pccn*	12.038	11.097	11.793	90	90	90	-217932.46	0.736
3421	*P*4$_2$/*n*	15.831	15.831	6.339	90	90	90	-217932.39	0.723
100	*P*2$_1$/*n*	3.925	8.691	22.470	90	93.72	90	-217932.31	0.758
1272	*P*2$_1$/*n*	8.486	4.373	11.014	90	110.00	90	-217932.23	0.754
1292	*P*2$_1$/*c*	9.014	11.440	7.510	90	94.16	90	-217932.09	0.748
175	*P*2$_1$/*c*	3.972	9.205	10.430	90	91.66	90	-217931.97	0.754

Str.	Space group	Cell parameters						E_{CASTEP}/ kJ mol^{-1}	PI
		a/Å	*b*/Å	*c*/Å	*α*/°	*β*/°	*γ*/°		
525	$P2_1/n$	8.183	10.710	8.959	90	100.81	90	-217931.75	0.750
47	*C*2/*c*	31.017	4.439	11.297	90	96.48	90	-217931.48	0.750
5122	*P*-1	4.492	11.239	16.319	109.23	97.15	92.00	-217931.38	0.754
6006	*C*2/*c*	31.146	4.378	11.293	90	96.48	90	-217931.30	0.761
427	*C*2/*c*	15.840	4.396	11.211	90	102.66	90	-217931.28	0.765
70	$P2_1/c$	7.266	9.867	11.064	90	102.21	90	-217930.80	0.745
526	*C*2/*c*	15.643	4.496	22.176	89.70	101.92	90.36	-217930.56	0.758
65	$Pca2_1$	17.523	3.954	10.863	90	90	90	-217930.50	0.773
1698	*C*2/*c*	13.551	9.562	12.389	89.66	98.32	89.83	-217930.44	0.725

Table S6. Computationally generated low-energy **4,4'-bipyridine** structures. The experimental structure is highlighted in green.

Str.	Space group	Cell parameters						E_{CASTEP}/ kJ mol^{-1}	PI
		a/Å	*b*/Å	*c*/Å	*α*/°	*β*/°	*γ*/°		
179	$P2_1/c$	9.265	10.580	8.188	90	103.50	90	-217927.72	0.743
1333	$P2_1$	3.777	10.278	10.226	90	92.00	90	-217927.38	0.731
1149	*Pbca*	7.302	11.309	18.242	90	90	90	-217927.34	0.770
2471	*P*-1	8.395	9.064	10.738	81.66	81.63	77.49	-217927.33	0.735
648b	*Pbca*	7.445	11.118	36.510	90	90	90	-217926.35	0.769
763	*I*2/*c*	7.433	5.743	35.512	90	94.59	90	-217925.91	0.766
7091	*P*2/*c*	14.527	3.720	29.355	90	90.01	90	-217925.88	0.723
379	$P2_1/c$	8.739	5.469	7.574	90	94.55	90	-217925.88	0.802
6367	*C*2/*c*	6.186	18.300	7.093	90.41	111.66	90.13	-217925.80	0.764
78	$P2_1/c$	9.672	7.410	11.249	90	109.86	90	-217925.78	0.765
333	*C*2/*c*	9.694	9.095	9.231	90	110.59	90	-217925.47	0.759
5529	*C*2/*c*	18.612	3.777	10.912	90	103.65	90	-217925.07	0.780
67	*P*-1	5.782	9.015	15.463	92.81	100.11	107.70	-217924.71	0.774
1874	*P*-1	3.828	9.388	21.535	80.69	86.52	81.13	-217924.62	0.766
771	*I*2/*c*	7.335	5.613	18.279	90	100.50	90	-217924.60	0.781
1476	*C*2/*c*	11.827	5.775	11.403	90	104.20	90	-217924.52	0.768
8319	*I*2	3.772	5.620	17.727	90	95.25	90	-217924.18	0.774
9284	*Pbca*	15.001	11.070	18.198	90	90	90	-217924.12	0.768
1631	$P2_1/n$	9.615	7.210	22.132	90	95.96	90	-217924.11	0.759
8367	*P*-1	6.515	11.310	11.988	109.19	100.85	103.97	-217923.95	0.751
7291	*P*-1	3.789	10.870	18.093	89.88	89.98	88.98	-217923.87	0.776
84	*Pbcn*	5.762	18.297	7.154	90	90	90	-217923.82	0.762
312	$P2_1/n$	8.162	5.529	16.361	90	91.82	90	-217923.79	0.762
692	*I*2/*a*	7.297	5.763	17.823	90.00	98.06	90	-217923.78	0.784
3170	$P2_1/n$	10.569	9.591	15.242	90	99.15	90	-217923.54	0.762
756	$P2_1/c$	9.568	10.036	9.062	90	117.36	90	-217923.45	0.754
125	$P2_1/n$	3.828	5.440	17.804	90	94.81	90	-217923.45	0.778
1532	$C222_1$	5.587	18.386	7.213	90	90	90	-217923.34	0.778
862	*Pbca*	18.286	7.257	22.938	90	90	90	-217923.06	0.763
891	*Pccn*	36.201	5.814	7.135	90	90	90	-217923.00	0.773
268	$P2_1/n$	10.339	5.986	13.065	90	110.71	90	-217922.89	0.768
8547	*Ibca*	5.812	7.132	36.634	90	90	90	-217922.84	0.761
7646	*P*1	12.273	3.877	15.941	89.89	97.98	89.87	-217922.70	0.767
6662	*P*-1	6.675	7.408	8.905	106.10	110.21	95.52	-217922.69	0.739
6531	$P2_1/c$	3.762	5.613	35.557	90	90.16	90	-217922.42	0.770
8415	*Pbcn*	5.699	7.321	17.983	90	90	90	-217922.33	0.770

Str.	Space group	Cell parameters						E_{CASTEP}	PI
		a/Å	*b*/Å	*c*/Å	*α*/°	*β*/°	*γ*/°	kJ mol^{-1}	
1383	*C*2/*c*	18.185	5.676	14.747	90	100.73	90	-217922.30	0.773
2534	*C*2/*c*	10.097	5.925	50.970	90	95.22	90	-217922.22	0.766
2249	*Pbca*	18.347	7.252	23.099	90	90	90	-217921.87	0.752
1313	*P*-1	3.832	11.791	17.161	80.39	84.68	83.14	-217921.75	0.764
6289	*P*-1	5.844	9.886	27.721	80.61	87.79	76.43	-217921.59	0.757
7077	$P2_1/c$	18.070	10.518	8.025	89.80	91.25	90.31	-217921.45	0.763
2328	*P*-1	5.423	11.576	12.146	97.22	90.45	90.95	-217921.43	0.760
6407	*P*-1	7.343	9.516	22.137	97.67	90.28	90.94	-217921.12	0.755
5162	*P*2/*c*	16.965	5.792	15.695	90	92.45	90	-217920.94	0.752
4877	$P2_1/c$	14.128	5.943	36.544	90	91.39	90	-217920.52	0.755
8631	$P2_1/c$	36.173	5.769	7.136	90	90.31	90	-217920.33	0.778
4916	*Pbca*	7.193	11.885	36.226	90	90	90	-217920.16	0.745
537	*C*2/*c*	17.013	5.731	17.435	89.38	115.88	90.64	-217920.06	0.758
1684	*P*2/*n*	11.343	5.785	11.946	90	103.09	90	-217919.19	0.761
4244	*Pn*	12.796	7.221	17.122	90	102.69	90	-217918.61	0.757

1.5. Computational generation of dapsone:2,2'-bipyridine (1:1) low-energy structures

Table S7. Computationally generated low-energy **dapsone:2,2'-bipyridine** structures. The experimental structure is highlighted in green.

Str.	Space group	Cell parameters						$\Delta\Delta E_F^{CC}$ [a]/	PI
		a/Å	*b*/Å	*c*/Å	*α*/°	*β*/°	*γ*/°	kJ mol^{-1}	
22-1	$P2_1$	8.200	16.120	14.602	90	99.64	90	-11.6	0.737
22-2	$P2_1$	8.248	15.902	14.812	90	100.30	90	-9.2	0.733
22-3	$P2_12_12_1$	7.050	12.817	21.318	90	90	90	-6.6	0.725
22-4	$P2_1$	8.290	15.825	14.889	90	98.16	90	2.5	0.725
22-5	$Pna2_1$	41.554	5.749	8.063	90	90	90	2.8	0.732
22-6	$P2_1/c$	14.500	8.097	17.174	90	91.36	90	3.0	0.698
22-7	$P2_1/c$	14.857	7.873	17.019	90	91.38	90	4.7	0.708
22-8	$P2_1/c$	13.927	8.190	17.419	90	96.79	90	4.7	0.715
22-9	$P2_1$	5.615	7.835	21.674	90	93.69	90	4.9	0.744
22-10	$P2_1/c$	16.935	7.721	17.127	90	118.77	90	4.9	0.718
22-11	P-1	8.217	8.959	13.764	88.21	89.24	77.34	6.0	0.709
22-12	$P2_1/c$	10.142	13.825	14.585	90	106.27	90	6.0	0.721
22-13	$P2_1$/n	6.008	7.765	42.204	90	91.86	90	7.1	0.715
22-14	$P2_1/c$	14.677	7.792	17.190	90	91.66	90	7.3	0.714
22-15	$P2_12_12_1$	6.295	13.891	22.459	90	90	90	7.9	0.722
22-16	$P2_12_12_1$	6.019	13.648	23.673	90	90	90	8.1	0.726
22-17	$P2_1/c$	13.789	8.119	17.712	90	94.37	90	8.3	0.711
22-18	P-1	8.233	9.061	14.039	84.88	79.31	70.89	8.7	0.724
22-19	$P2_1/n$	6.304	14.667	21.053	90	92.60	90	9.0	0.728
22-20	$P2_1/c$	14.268	13.417	10.114	90	92.01	90	9.4	0.731
22-21	$P2_1/c$	19.716	6.443	15.670	90	100.33	90	11.2	0.718
22-22	$P2_1/n$	6.308	13.597	23.140	90	95.56	90	12.5	0.714
22-23	$P2_12_12_1$	6.075	13.527	23.793	90	90	90	12.6	0.726
22-24	$P2_12_12_1$	6.106	14.193	22.431	90	90	90	12.8	0.728
22-25	$P2_1/n$	6.451	14.167	21.539	90	93.70	90	12.9	0.719
22-26	$P2_1/n$	6.308	14.255	21.701	90	97.58	90	14.2	0.729
22-27	$Pna2_1$	21.603	14.182	6.263	90	90	90	15.2	0.740
22-28	$P2_1/c$	6.277	14.441	21.857	90	96.04	90	16.2	0.717
22-29	$P2_1/n$	6.311	20.697	14.723	90	94.96	90	16.3	0.737
22-30	$P2_1/n$	6.244	15.642	20.053	90	96.42	90	18.6	0.726
22-31	$P2_1/n$	6.404	14.473	21.287	90	96.17	90	18.9	0.719
22-32	$C2/c$	26.850	8.381	17.900	90	94.36	90	21.0	0.701

[a] calculated according to equ. (5) of the manuscript.

1.6. Computational generation of dapsone:4,4’-bipyridine (1:1) low-energy structures

Table S8. Computationally generated low-energy **dapsone**:**4,4’-bipyridine** structures. The experimental structures are highlighted in green.

Str.	Space group	Cell parameters						$\Delta\Delta E_F^{CC}$ [a] / kJ mol⁻¹	PI
		a/Å	*b*/Å	*c*/Å	*α*/°	*β*/°	*γ*/°		
CC_{44}-B	$P2_1/n$	5.816	7.652	33.885	90.00	91.31	90.00	-19.5	0.746
44-1	$P2_1$	5.731	7.727	21.293	90	96.65	90	-14.0	0.754
44-2	$Pca2_1$	42.575	5.696	7.718	90	90	90	-13.1	0.754
44-3	$P2_1/n$	5.772	7.712	42.305	90	93.78	90	-12.2	0.750
44-4	$P2_12_12_1$	5.747	7.640	42.998	90	90	90	-11.9	0.749
44-5	$P2_1/c$	12.369	7.887	20.200	89.51	106.19	89.88	-11.0	0.745
44-6	$P2_12_12$	7.799	41.756	5.791	90	90	90	-9.8	0.750
44-7	$Pna2_1$	43.651	5.650	7.566	90	90	90	-7.1	0.754
44-8	$P2_1/c$	9.544	7.887	25.905	90	99.04	90	-6.5	0.734
44-9	$Pbca$	12.450	16.475	19.393	90	90	90	-5.3	0.705
44-10	$P2_12_12_1$	5.772	8.007	40.340	90	90	90	-3.9	0.760
44-11	P-1	9.528	13.457	16.270	88.88	89.62	80.81	-2.4	0.676
44-12	$P2_1$	5.787	22.546	14.538	90	90.31	90	-1.0	0.740
44-13	$P2_1/n$	8.194	18.684	14.482	90	105.84	90	0.3	0.656
44-14	$P2_1/n$	15.185	7.402	18.710	89.79	107.48	89.49	1.2	0.702
44-15	$P2_12_12_1$	5.891	10.967	29.421	90	90	90	2.8	0.740
44-16	$P2_1/n$	11.106	15.230	12.172	90	90.72	90	3.1	0.685
44-17	Cc	6.229	41.267	7.523	89.80	91.49	90.44	3.7	0.732
44-18	$P2_1/c$	13.553	7.611	20.409	90	104.66	90	5.8	0.690
44-19	$P2_1/c$	9.757	13.387	15.216	90	106.12	90	6.0	0.744
44-20	$P2_1/n$	5.944	16.138	20.149	90	98.19	90	9.8	0.736
44-22	$P2_1/c$	5.628	7.769	42.909	90	91.65	90	10.4	0.752
44-23	$P2_1/n$	6.505	14.191	21.144	90	91.19	90	11.0	0.726
44-24	$P2_1/n$	10.257	13.157	14.830	89.54	106.38	90.36	11.3	0.738

[a] calculated according to equ. (5) of the manuscript.

2. Experimental Dapsone:2,2’-Bipyridine cocrystal screen

2.1. Grinding experiments (dry and liquid-assisted)

Table S9. Dapsone:2,2’-Bipyridine grinding experiments: DDS-III – dapsone form III, DDS-V – dapsone form V, DDS-H – dapsone hydrate, 2,2’BP – 2,2’-bipyridine, CC-A – dapsone:2,2’-bipyridine cocrystal A, CC-B – dapsone:2,2’-bipyridine cocrystal B.

Solvent	Dapsone / mg	2,2‘-BIPY / mg	Ratio [DAP:BIPY]	Time / min	Solid-state form					
					DDS-III	DDS-V	DDS-H	2,2’BP	CC-A	CC-B
t-BuOH	92.71	57.51	1:1	0	X			X		
t-BuOH	92.71	57.51	1:1	5	X			X	X	X
t-BuOH	92.71	57.51	1:1	10	X*					X
t-BuOH	92.71	57.51	1:1	15	X*					X
t-BuOH	92.71	57.51	1:1	30	X*					X
t-BuOH	92.71	57.51	1:1	45		X*				X
t-BuOH	92.71	57.51	1:1	60		X*				X
t-BuOH	66.43	83.91	1:2	0	X			X		
t-BuOH	66.43	83.91	1:2	5				X		X
t-BuOH	66.43	83.91	1:2	10				X		X
t-BuOH	66.43	83.91	1:2	15				X		X
t-BuOH	66.43	83.91	1:2	30				X		X
t-BuOH	66.43	83.91	1:2	45				X		X
t-BuOH	66.43	83.91	1:2	60				X		X
t-BuOH	113.81	35.43	2:1	0	X			X		
t-BuOH	113.81	35.43	2:1	5	X				X	
t-BuOH	113.81	35.43	2:1	10	X				X	
t-BuOH	113.81	35.43	2:1	15	X				X	
t-BuOH	113.81	35.43	2:1	30		X			X	
t-BuOH	113.81	35.43	2:1	45		X			X	
t-BuOH	113.81	35.43	2:1	60		X			X	X
i-BuOAc	92.16	57.45	1:1	0	X			X		
i-BuOAc	92.16	57.45	1:1	5	X*					X
i-BuOAc	92.16	57.45	1:1	10	X*					X
i-BuOAc	92.16	57.45	1:1	15	X*					X
i-BuOAc	92.16	57.45	1:1	30		X*				X
i-BuOAc	92.16	57.45	1:1	45		X*				X
i-BuOAc	92.16	57.45	1:1	60		X*				X
i-BuOAc	66.61	83.33	1:2	0	X			X		
i-BuOAc	66.61	83.33	1:2	5				X		X
i-BuOAc	66.61	83.33	1:2	10				X		X
i-BuOAc	66.61	83.33	1:2	15				X		X
i-BuOAc	66.61	83.33	1:2	30				X		X
i-BuOAc	66.61	83.33	1:2	45				X		X
i-BuOAc	66.61	83.33	1:2	60				X		X
i-BuOAc	114.36	36.21	2:1	0	X					
i-BuOAc	114.36	36.21	2:1	5	X				X	X
i-BuOAc	114.36	36.21	2:1	10	X					X
i-BuOAc	114.36	36.21	2:1	15	X					X
i-BuOAc	114.36	36.21	2:1	30		X				X
i-BuOAc	114.36	36.21	2:1	45		X				X
i-BuOAc	114.36	36.21	2:1	60		X				X

Solvent	Dapsone / mg	2,2‘-BIPY / mg	Ratio [DAP:BIPY]	Time / min	Solid-state form					
					DDS-III	DDS-V	DDS-H	2,2’BP	CC-A	CC-B
DIPE	92.1	59.7	1:1	0	X			X		
DIPE	92.1	59.7	1:1	5					X	X*
DIPE	92.1	59.7	1:1	10					X	X
DIPE	92.1	59.7	1:1	15					X	X
DIPE	92.1	59.7	1:1	30					X	X
DIPE	92.1	59.7	1:1	45					X	X
DIPE	92.1	59.7	1:1	60					X	X
DIPE	66.38	83.52	1:2	0	X			X		
DIPE	66.38	83.52	1:2	5				X	X	X*
DIPE	66.38	83.52	1:2	10				X	X*	X
DIPE	66.38	83.52	1:2	15				X		X
DIPE	66.38	83.52	1:2	30				X		X
DIPE	66.38	83.52	1:2	45				X		X
DIPE	66.38	83.52	1:2	60				X		X
DIPE	114.37	35.60	2:1	0	X			X		
DIPE	114.37	35.60	2:1	5	X				X	X
DIPE	114.37	35.60	2:1	10	X				X	X
DIPE	114.37	35.60	2:1	15	X				X	X
DIPE	114.37	35.60	2:1	30	X				X	X
DIPE	114.37	35.60	2:1	45	X				X	X
DIPE	114.37	35.60	2:1	60	X				X	X
water	92.15	58.10	1:1	0	X			X		
water	92.15	58.10	1:1	5					X	
water	92.15	58.10	1:1	10					X	
water	92.15	58.10	1:1	15					X	
water	92.15	58.10	1:1	30					X	
water	92.15	58.10	1:1	45					X	
water	92.15	58.10	1:1	60					X	
water	66.50	86.60	1:2	0	X			X		
water	66.50	86.60	1:2	5				X	X	X*
water	66.50	86.60	1:2	10				X	X*	X
water	66.50	86.60	1:2	15				X		X
water	66.50	86.60	1:2	30				X		X
water	66.50	86.60	1:2	45				X		X
water	66.50	86.60	1:2	60				X		X
water	114.09	35.85	2:1	0	X			X		
water	114.09	35.85	2:1	5	X					
water	114.09	35.85	2:1	10			X		X	
water	114.09	35.85	2:1	15			X		X	
water	114.09	35.85	2:1	30			X		X	
water	114.09	35.85	2:1	45			X		X	
water	114.09	35.85	2:1	60			X		X	
n-heptane	92.03	58.02	1:1	0	X			X		
n-heptane	92.03	58.02	1:1	5	X			X	X	
n-heptane	92.03	58.02	1:1	10	X			X	X	X*
n-heptane	92.03	58.02	1:1	15	X			X*	X	X
n-heptane	92.03	58.02	1:1	30	X*				X	X
n-heptane	92.03	58.02	1:1	45	X*				X	X
n-heptane	92.03	58.02	1:1	60	X*				X	X

Solvent	Dapsone / mg	2,2‘-BIPY / mg	Ratio [DAP:BIPY]	Time / min	Solid-state form					
					DDS-III	DDS-V	DDS-H	2,2’BP	CC-A	CC-B
n-heptane	66.51	83.55	1:2	0	X			X		
n-heptane	66.51	83.55	1:2	5				X	X	
n-heptane	66.51	83.55	1:2	10				X	X	X*
n-heptane	66.51	83.55	1:2	15				X	X	X*
n-heptane	66.51	83.55	1:2	30				X	X*	X
n-heptane	66.51	83.55	1:2	45				X		X
n-heptane	66.51	83.55	1:2	60				X		X
n-heptane	114.12	35.90	2:1	0	X			X		
n-heptane	114.12	35.90	2:1	5	X				X	
n-heptane	114.12	35.90	2:1	10	X				X	
n-heptane	114.12	35.90	2:1	15	X				X	
n-heptane	114.12	35.90	2:1	30	X				X	
n-heptane	114.12	35.90	2:1	45	X				X	
n-heptane	114.12	35.90	2:1	60	X				X	
dry	122.44	77.16	1:1	0	X			X		
dry	122.44	77.16	1:1	5	X			X		
dry	122.44	77.16	1:1	10	X			X	X	
dry	122.44	77.16	1:1	15	X*			X*	X	
dry	122.44	77.16	1:1	30					X	
dry	122.44	77.16	1:1	45					X	
dry	122.44	77.16	1:1	60					X	
dry	88.43	111.33	1:2	0	X			X		
dry	88.43	111.33	1:2	5	X			X		
dry	88.43	111.33	1:2	10	X			X	X*	
dry	88.43	111.33	1:2	15	X*			X	X	
dry	88.43	111.33	1:2	30				X	X	
dry	88.43	111.33	1:2	45				X	X	
dry	88.43	111.33	1:2	60				X	X	
dry	152.00	47.56	2:1	0	X			X		
dry	152.00	47.56	2:1	5	X			X		
dry	152.00	47.56	2:1	10	X			X	X	
dry	152.00	47.56	2:1	15	X			X	X	
dry	152.00	47.56	2:1	30	X				X	
dry	152.00	47.56	2:1	45	X				X	
dry	152.00	47.56	2:1	60	X				X	

*traces of the solid-state form are present.

2.2. Slurry experiments in organic solvents

Table S10. Dapsone:2,2’-Bipyridine slurry experiments: DDS-III – dapsone form III, DDS-V – dapsone form V, DDS-H – dapsone hydrate, 2,2’BP – 2,2’-bipyridine, CC-A – dapsone:2,2’-bipyridine cocrystal A, CC-B – dapsone:2,2’-bipyridine cocrystal B.

Solvent	Dapsone / mg	2,2‘-BIPY / mg	Ratio [DAP:BIPY]	Time / days	Solid-state form					
					DDS-III	DDS-V	DDS-H	2,2’BP	CC-A	CC-B
DIPE	92.12	58.11	1:1	0	X			X		
DIPE	92.12	58.11	1:1	1				X	X	X*
DIPE	92.12	58.11	1:1	2				X	X	X*
DIPE	92.12	58.11	1:1	3				X	X	X*
DIPE	92.12	58.11	1:1	7				X		X
DIPE	66.49	83.51	1:2	0	X			X		
DIPE	66.49	83.51	1:2	1				X	X	
DIPE	66.49	83.51	1:2	2				X	X	
DIPE	66.49	83.51	1:2	3				X	X	
DIPE	66.49	83.51	1:2	7				X	X*	X
DIPE	114.09	35.61	2:1	0	X			X		
DIPE	114.09	35.61	2:1	1				X*	X	
DIPE	114.09	35.61	2:1	2				X*	X	
DIPE	114.09	35.61	2:1	3				X*	X	
DIPE	114.09	35.61	2:1	7				X*		X
water	92.23	58.15	1:1	0	X			X		
water	92.23	58.15	1:1	1					X	
water	92.23	58.15	1:1	2					X	
water	92.23	58.15	1:1	3					X	
water	92.23	58.15	1:1	7					X	
water	66.49	83.23	1:2	0	X			X		
water	66.49	83.23	1:2	1				X	X	
water	66.49	83.23	1:2	2				X	X	
water	66.49	83.23	1:2	3				X	X	
water	66.49	83.23	1:2	7				X	X	
water	114.28	36.04	2:1	0	X			X		
water	114.28	36.04	2:1	1			X		X	
water	114.28	36.04	2:1	2			X		X	
water	114.28	36.04	2:1	3			X		X	
water	114.28	36.04	2:1	7			X		X	
n-heptane	92.12	58.11	1:1	0	X			X		
n-heptane	92.12	58.11	1:1	1	X			X	X	X
n-heptane	92.12	58.11	1:1	2				X*	X	X
n-heptane	92.12	58.11	1:1	3				X*	X	X
n-heptane	92.12	58.11	1:1	7				X*		X
n-heptane	66.70	83.27	1:2	0	X			X		
n-heptane	66.70	83.27	1:2	1				X	X	X
n-heptane	66.70	83.27	1:2	2				X	X	X
n-heptane	66.70	83.27	1:2	3				X	X	X
n-heptane	66.70	83.27	1:2	7				X	X*	X
n-heptane	114.29	35.96	2:1	0	X			X		
n-heptane	114.29	35.96	2:1	1	X			X*	X	X
n-heptane	114.29	35.96	2:1	2	X			X*	X	X
n-heptane	114.29	35.96	2:1	3	X			X*	X	X
n-heptane	114.29	35.96	2:1	7	X			X*	X	X

*traces of the solid-state form are present.

3. Experimental Dapsone:4,4'-Bipyridine cocrystal screen

3.1. Grinding experiments (dry and liquid-assisted)

Table S11. Dapsone:4,4'-Bipyridine grinding experiments: DDS-III – dapsone form III, DDS-V – dapsone form V, DDS-H – dapsone hydrate, 4,4'BP-A – 4,4'-bipyridine anhydrate, 4,4'BP-H – 4,4'-bipyridine hydrate, CC-A – dapsone:4,4'-bipyridine cocrystal A, CC-B – dapsone:4,4'-bipyridine cocrystal B.

Solvent	Dapsone / mg	4,4'-BIPY / mg	Ratio [DAP:BIPY]	Time / min	Solid-state form					
					DDS-III/V	DDS-H	4,4'BP-A	4,4'BP-H	CC-A	CC-B
t-BuOH	122.68	77.23	1:1	0	X		X			
t-BuOH	122.68	77.23	1:1	5	X					
t-BuOH	122.68	77.23	1:1	10	X					
t-BuOH	122.68	77.23	1:1	15					X	
t-BuOH	122.68	77.23	1:1	30					X	
t-BuOH	122.68	77.23	1:1	45					X	
t-BuOH	122.68	77.23	1:1	60					X	
t-BuOH	66.26	83.41	1:2	0	X		X			
t-BuOH	66.26	83.41	1:2	5			X		X	
t-BuOH	66.26	83.41	1:2	10			X		X	
t-BuOH	66.26	83.41	1:2	15			X		X	
t-BuOH	66.26	83.41	1:2	30			X		X	
t-BuOH	66.26	83.41	1:2	45			X		X	
t-BuOH	66.26	83.41	1:2	60			X		X	
t-BuOH	113.97	35.59	2:1	0	X		X			
t-BuOH	113.97	35.59	2:1	5	X*					X
t-BuOH	113.97	35.59	2:1	10						X
t-BuOH	113.97	35.59	2:1	15						X
t-BuOH	113.97	35.59	2:1	30						X
t-BuOH	113.97	35.59	2:1	45						X
t-BuOH	113.97	35.59	2:1	60						X
i-BuOAc	92.11	57.61	1:1	0	X		X			
i-BuOAc	92.11	57.61	1:1	5					X	
i-BuOAc	92.11	57.61	1:1	10					X	
i-BuOAc	92.11	57.61	1:1	15					X	
i-BuOAc	92.11	57.61	1:1	30					X	
i-BuOAc	92.11	57.61	1:1	45					X	
i-BuOAc	92.11	57.61	1:1	60					X	
i-BuOAc	66.37	83.21	1:2	0	X		X			
i-BuOAc	66.37	83.21	1:2	5			X		X	
i-BuOAc	66.37	83.21	1:2	10			X		X	
i-BuOAc	66.37	83.21	1:2	15			X		X	
i-BuOAc	66.37	83.21	1:2	30			X		X	
i-BuOAc	66.37	83.21	1:2	45			X		X	
i-BuOAc	66.37	83.21	1:2	60			X		X	
i-BuOAc	114.21	35.87	2:1	0	X		X			
i-BuOAc	114.21	35.87	2:1	5						X
i-BuOAc	114.21	35.87	2:1	10						X
i-BuOAc	114.21	35.87	2:1	15						X
i-BuOAc	114.21	35.87	2:1	30						X
i-BuOAc	114.21	35.87	2:1	45						X
i-BuOAc	114.21	35.87	2:1	60						X

Solvent	Dapsone / mg	4,4'-BIPY / mg	Ratio [DAP:BIPY]	Time / min	Solid-state form					
					DDS-III/V	DDS-H	4,4'BP-A	4,4'BP-H	CC-A	CC-B
DIPE	92.11	58.08	1:1	0	X		X			
DIPE	92.11	58.08	1:1	5	X		X		X*	
DIPE	92.11	58.08	1:1	10	X		X		X	
DIPE	92.11	58.08	1:1	15	X*		X*		X	
DIPE	92.11	58.08	1:1	30	X*		X*		X	
DIPE	92.11	58.08	1:1	45	X*		X*		X	
DIPE	92.11	58.08	1:1	60					X	
DIPE	66.64	83.35	1:2	0	X		X			
DIPE	66.64	83.35	1:2	5	X		X		X*	
DIPE	66.64	83.35	1:2	10	X		X		X	
DIPE	66.64	83.35	1:2	15	X*		X		X	
DIPE	66.64	83.35	1:2	30	X*		X		X	
DIPE	66.64	83.35	1:2	45	X*		X		X	
DIPE	66.64	83.35	1:2	60			X		X	
DIPE	113.93	35.61	2:1	0	X		X			
DIPE	113.93	35.61	2:1	5	X				X	X
DIPE	113.93	35.61	2:1	10	X				X	X
DIPE	113.93	35.61	2:1	15	X*				X*	X
DIPE	113.93	35.61	2:1	30						X
DIPE	113.93	35.61	2:1	45						X
DIPE	113.93	35.61	2:1	60						X
water	92.11	57.71	1:1	0	X		X			
water	92.11	57.71	1:1	5	X			X		X
water	92.11	57.71	1:1	10	X*			X		X
water	92.11	57.71	1:1	15				X		X
water	92.11	57.71	1:1	30				X		X
water	92.11	57.71	1:1	45				X		X
water	92.11	57.71	1:1	60				X		X
water	66.37	83.74	1:2	0	X		X			
water	66.37	83.74	1:2	5	X			X		X
water	66.37	83.74	1:2	10	X			X		X
water	66.37	83.74	1:2	15	X*			X		X
water	66.37	83.74	1:2	30				X		X
water	66.37	83.74	1:2	45				X		X
water	66.37	83.74	1:2	60				X		X
water	114.32	35.97	2:1	0	X		X			
water	114.32	35.97	2:1	5	X			X		X
water	114.32	35.97	2:1	10	X*			X*		X
water	114.32	35.97	2:1	15	X*			X*		X
water	114.32	35.97	2:1	30						X
water	114.32	35.97	2:1	45						X
water	114.32	35.97	2:1	60						X
n-heptane	92.18	58.01	1:1	0	X		X			
n-heptane	92.18	58.01	1:1	5	X		X		X*	
n-heptane	92.18	58.01	1:1	10	X		X		X	
n-heptane	92.18	58.01	1:1	15	X		X		X	
n-heptane	92.18	58.01	1:1	30	X*		X*		X	
n-heptane	92.18	58.01	1:1	45					X	
n-heptane	92.18	58.01	1:1	60					X	

Solvent	Dapsone / mg	4,4‘-BIPY / mg	Ratio [DAP:BIPY]	Time / min	Solid-state form					
					DDS-III/V	DDS-H	4,4’BP-A	4,4’BP-H	CC-A	CC-B
n-heptane	66.49	83.59	1:2	0	X		X			
n-heptane	66.49	83.59	1:2	5	X		X		X	
n-heptane	66.49	83.59	1:2	10	X		X		X	
n-heptane	66.49	83.59	1:2	15	X		X		X	
n-heptane	66.49	83.59	1:2	30	X*		X		X	
n-heptane	66.49	83.59	1:2	45			X		X	
n-heptane	66.49	83.59	1:2	60			X		X	
n-heptane	114.06	36.05	2:1	0	X		X			
n-heptane	114.06	36.05	2:1	5	X				X	X
n-heptane	114.06	36.05	2:1	10	X				X	X
n-heptane	114.06	36.05	2:1	15	X				X	X
n-heptane	114.06	36.05	2:1	30	X*				X*	X
n-heptane	114.06	36.05	2:1	45	X*				X*	X
n-heptane	114.06	36.05	2:1	60						X
dry	92.34	58.17	1:1	0	X		X			
dry	92.34	58.17	1:1	5	X		X		X	X
dry	92.34	58.17	1:1	10	X		X		X	X
dry	92.34	58.17	1:1	15	X		X		X	X
dry	92.34	58.17	1:1	30			X*		X	X*
dry	92.34	58.17	1:1	45			X*		X	X*
dry	92.34	58.17	1:1	60					X	
dry	66.48	83.60	1:2	0	X		X			
dry	66.48	83.60	1:2	5	X		X			X
dry	66.48	83.60	1:2	10	X		X			X
dry	66.48	83.60	1:2	15	X		X			X
dry	66.48	83.60	1:2	30	X*		X			X
dry	66.48	83.60	1:2	45			X		X	X*
dry	66.48	83.60	1:2	60			X		X	
dry	114.08	35.69	2:1	0	X		X			
dry	114.08	35.69	2:1	5	X		X			X
dry	114.08	35.69	2:1	10	X		X			X
dry	114.08	35.69	2:1	15	X		X			X
dry	114.08	35.69	2:1	30	X*		X*			X
dry	114.08	35.69	2:1	45	X*		X*			X
dry	114.08	35.69	2:1	60						X

*traces of the solid-state form are present.

3.2. Slurry experiments in organic solvents

Table S12. Dapsone:4,4’-Bipyridine slurry experiments: DDS-III – dapsone form III, DDS-V – dapsone form V, DDS-H – dapsone hydrate, 4,4’BP-A – 4,4’-bipyridine anhydrate, 4,4’BP-H – 4,4’-bipyridine hydrate, CC-A – dapsone:4,4’-bipyridine cocrystal A, CC-B – dapsone:4,4’-bipyridine cocrystal B.

Solvent	Dapsone / mg	4,4‘-BIPY / mg	Ratio [DAP:BIPY]	Time / days	Solid-state form					
					DDS-III/V	DDS-H	4,4’BP-A	4,4’BP-H	CC-A	CC-B
DIPE	92.29	58.00	1:1	0	X		X			
DIPE	92.29	58.00	1:1	1	X		X		X	
DIPE	92.29	58.00	1:1	2					X	
DIPE	92.29	58.00	1:1	3					X	
DIPE	92.29	58.00	1:1	7					X	
DIPE	66.38	83.40	1:2	0	X		X			
DIPE	66.38	83.40	1:2	1			X		X	
DIPE	66.38	83.40	1:2	2			X		X	
DIPE	66.38	83.40	1:2	3			X		X	
DIPE	66.38	83.40	1:2	7			X		X	
DIPE	114.31	36.02	2:1	0	X		X			
DIPE	114.31	36.02	2:1	1	X				X	X
DIPE	114.31	36.02	2:1	2	X				X	X
DIPE	114.31	36.02	2:1	3	X				X	X
DIPE	114.31	36.02	2:1	7	X*					X
water	92.02	57.91	1:1	0	X		X			
water	92.02	57.91	1:1	1	X			X		X
water	92.02	57.91	1:1	2	X*			X		X
water	92.02	57.91	1:1	3	X*			X		X
water	92.02	57.91	1:1	7				X		X
water	66.52	83.59	1:2	0	X		X			
water	66.52	83.59	1:2	1	X			X		X
water	66.52	83.59	1:2	2	X*			X		X
water	66.52	83.59	1:2	3				X		X
water	66.52	83.59	1:2	7				X		X
water	113.96	36.18	2:1	0	X		X			
water	113.96	36.18	2:1	1	X*			X*		X
water	113.96	36.18	2:1	2	X*			X*		X
water	113.96	36.18	2:1	3						X
water	113.96	36.18	2:1	7						X
n-heptane	92.22	57.81	1:1	0	X		X			
n-heptane	92.22	57.81	1:1	1					X	
n-heptane	92.22	57.81	1:1	2					X	
n-heptane	92.22	57.81	1:1	3					X	
n-heptane	92.22	57.81	1:1	7					X	
n-heptane	66.50	83.42	1:2	0	X		X			
n-heptane	66.50	83.42	1:2	1	X		X		X	
n-heptane	66.50	83.42	1:2	2	X*		X		X	
n-heptane	66.50	83.42	1:2	3	X*		X		X	
n-heptane	66.50	83.42	1:2	7			X		X	
n-heptane	113.97	35.78	2:1	0	X		X			
n-heptane	113.97	35.78	2:1	1	X				X	X
n-heptane	113.97	35.78	2:1	2	X				X	X
n-heptane	113.97	35.78	2:1	3	X				X	X
n-heptane	113.97	35.78	2:1	7	X				X	X

*traces of the solid-state form are present.

4. Characterization of the cocrystals

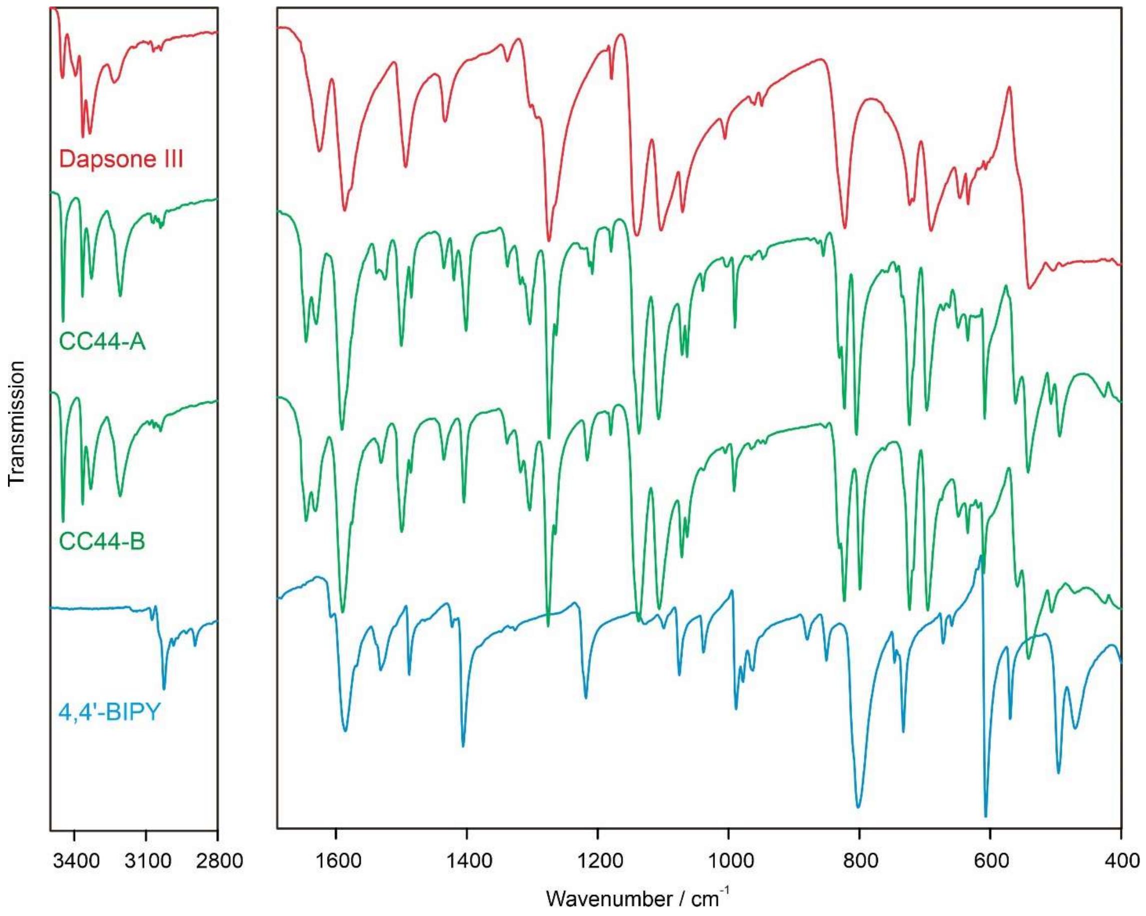

Figure S2. IR spectra of DDS (forms III), DDS:4,4'-BIPY cocrystals (**CC$_{44}$-A** and **CC$_{44}$-B**) and 4,4'-BIPY.

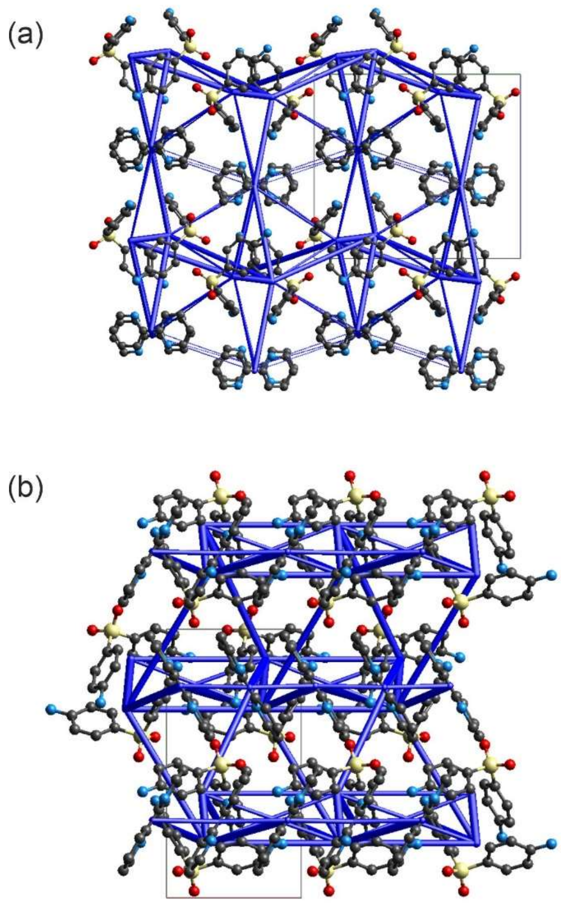

Figure S3. Energy framework diagram (total energy) for the **CC$_{22}$-A** cocrystal. The energy scale factor is 60. Stabilizing contacts are shown in blue and the thickness corresponds to the strength. Pairwise interaction energies <5 and <20 kJ mol^{-1} are omitted in (a) and (b), respectively. (a) Viewed along the *a* and (b) along the *c* crystallographic axis.

References

(1) Macrae, C. F.; Sovago, I.; Cottrell, S. J.; Galek, P. T. A.; McCabe, P.; Pidcock, E.; Platings, M.; Shields, G. P.; Stevens, J. S.; Towler, M.; Wood, P. A., Mercury 4.0: from visualization to analysis, design and prediction. *Journal of Applied Crystallography* **2020,** 53, (1), 226-235.
(2) Braun, D. E.; Vickers, M.; Griesser, U. J., Dapsone Form V: A Late Appearing Thermodynamic Polymorph of a Pharmaceutical. *Molecular Pharmaceutics* **2019,** 16, 3221-3236.
(3) Braun, D. E.; Hald, P.; Kahlenberg, V.; Griesser, U. J., Expanding the Solid Form Landscape of Bipyridines. *Crystal Growth & Design* **2021,** 21, (12), 7201-7217.
